# GS2 cooperates with IPA1 to control panicle architecture

**DOI:** 10.1111/nph.20412

**Published:** 2025-01-31

**Authors:** Yueying Wang, Yang Lv, Yi Wen, Junge Wang, Peng Hu, Kaixiong Wu, Bingze Chai, Shuxian Gan, Jialong Liu, Yue Wu, Lixin Zhu, Nannan Dong, Yiqing Tan, Hao Wu, Guangheng Zhang, Li Zhu, Deyong Ren, Qiang Zhang, Yuexing Wang, Qian Qian, Jiang Hu

**Affiliations:** ^1^ State Key Laboratory of Rice Biology and Breeding China National Rice Research Institute Hangzhou 311401 China; ^2^ Institute of Agricultural Sciences, Xishuangbanna Prefecture Jinghong Yunnan Province 666100 China; ^3^ Academician Workstation, National Nanfan Research Institute (Sanya), Chinese Academy of Agricultural Sciences Sanya 572024 China; ^4^ Hainan Seed Industry Laboratory Sanya 572024 China

**Keywords:** grain number, miRNA, panicle architecture, rice, transcriptional regulation

## Abstract

- Panicle size and grain number are important agronomic traits that determine grain yield in rice. However, the underlying mechanism regulating panicle size and grain number remains largely unknown.
- Here, we report that *GS2* plays an important role in regulating panicle architecture. The RNAi of *GS2™* (target site mutation, TM) produced erect and dense panicle with increased primary and secondary branches and grain number per panicle, whereas the overexpression of *GS2™* showed longer panicles and fewer grains than wild‐type.
- GS2 directly binds to the GCCA motif and significantly enhances the transcriptional activation ability through the interaction with IPA1. *DEP1* is a common target gene of GS2 and IPA1 in regulating branch number and grain number per panicle. The pyramiding of *GS2™* and *IPA1™* ^*1*^ (Target site mutation1, TM1) on hybrid rice can significantly increase rice yield.
- Our findings reveal the novel function of *GS2* and the molecular mechanism of *GS2*/*IPA1*‐*DEP1* module in controlling panicle architecture.

## Introduction

As a staple food crop, rice is mainly cultivated in Asia and supports more than half of the world's population. Given the accelerating urbanization and continuing population growth, food security has received more and more attention world‐wide. Grain yield is the ultimate output of rice cultivation, which is closely associated with panicle number per plant, grain number per panicle and grain weight (Sakamoto & Matsuoka, 2008; Xing & Zhang, 2010). It is well known that breeding improvement of these three agronomic related traits is the most effective way to increase rice yield. Among them, the panicle architecture is crucial, which is mainly determined by panicle length, primary and secondary branches (SBs) (grain number) and grain size (grain weight) (Li & Li, 2016; Fan & Li, 2019; Li *et al*., 2019; Chun *et al*., 2022; Ren *et al*., 2023). Therefore, elucidating the molecular regulatory mechanism and genetic network of panicle architecture is necessary for high‐yield breeding in rice.

The panicle development is a sequential process regulated by a multigene network, which first converses from the shoot apical meristem (SAM) into the inflorescence meristem (IM); then, IM differentiates into the panicle axis, primary branch (PB) and SB primordia, and finally forms the spikelet meristem (SM) in rice (Ikeda *et al*., 2004; Itoh *et al*., 2005; Zhang & Yuan, 2014). Disruption of the process will lead to abnormal panicle development, such as lax or dense panicles, large or short panicles, frizzy panicles, no spikelet and even panicle degradation (Komatsu *et al*., 2003; Huang *et al*., 2009; Jiao *et al*., 2010; Tabuchi *et al*., 2011; Tanaka *et al*., 2015; Wang *et al*., 2018). To date, numerous quantitative trait loci (QTLs) or genes that affect rice panicle architecture have been identified by map‐based cloning and genome‐wide association studies (GWAS) strategies, which are mainly involved in ubiquitin–proteasome degradation, mitogen‐activated protein kinase (MAPK), G protein and phytohormone signaling, and transcriptional regulation pathways (Huang *et al*., 2009; Zhou *et al*., 2009; Huang *et al*., 2017; Wang *et al*., 2017; Guo *et al*., 2018; Guo *et al*., 2020; Li *et al*., 2021; Chen *et al*., 2022; Chun *et al*., 2023; Li *et al*., 2023; Wu *et al*., 2023).

The heterotrimeric G protein complex, which contains Gα, Gβ and Gγ subunit, plays a molecular switch role in signal transduction pathways (Xu *et al*., 2016; Ofoe, 2021). *DEP1* is a member of rice Gγ subunit, and its natural dominant variation has been shown to increase panicle branch and grain number per panicle, leading to an increase in grain yield (Huang *et al*., 2009; Zhou *et al*., 2009). Moreover, the dominant *dep1* allele also exhibits nitrogen‐insensitive vegetative growth, which increased harvest index and grain yield under moderate nitrogen fertilization conditions by enhancing nitrogen uptake and assimilation (Sun *et al*., 2014). Previous report has revealed that *DEP1* is a direct downstream target gene of IPA1 (Lu *et al*., 2013). *IPA1*/*OsSPL14* is a member of the SPL family of transcription factors and regulated by the *OsmiR156* (Lu *et al*., 2013). A point mutation of *OsSPL14* disrupts *OsmiR156*‐mediated transcript cleavage, generating an ‘ideal’ rice plant with reduced tiller number, increased culm strength, grain number per panicle and grain yield (Jiao *et al*., 2010; Miura *et al*., 2010). Therefore, the elite alleles of *DEP1* and *IPA1* have been widely used in *japonica* and *indica*‐*japonica* hybrid rice in China, respectively (Huang *et al*., 2009; Zhang *et al*., 2017). *GS2* ^*BDL*^, a rare allelic variation of *GS2*, is also a yield‐increasing gene and plays a positive regulatory role in grain size (Hu *et al*., 2015). Two base substitutions in growth‐regulating factor 4 (*OsGRF4*) encoded by *GS2* blocks *OsmiR396c*‐directed translation repression, thereby significantly improving yield by elevating grain size (Che *et al*., 2015; Duan *et al*., 2015; Hu *et al*., 2015). In addition, *GS2*/*GRF4* can also promote and integrate nitrogen assimilation, which increases rice yield by tilting the GRF4‐DELLA balance in the direction of increasing GRF4 abundance (Li *et al*., 2018). However, *GS2* ^*BDL*^/*GS2™* significantly reduces the appearance quality of rice, which limits the utilization of this gene in production (Hu *et al*., 2015).

Here, we characterized the RNA interference plants of *GS2™*, which exhibited an erect and dense panicle phenotype. Although *GS2* has been reported to participate in the control of panicle length and grain size, the molecular mechanism in regulating panicle branch and grain number remains unexplored. Our findings revealed that GS2 physically interacts with IPA1 to regulate panicle architecture by promoting the expression of the downstream target gene *DEP1*. Furthermore, the grain yield of hybrid rice pyramiding *GS*2*™* and *IPA1™* was significantly higher than that of containing only one of them. These findings reveal a previously unknown mechanism of panicle type and grain number development involving the GS2/IPA1‐DEP1 regulatory module in rice.

## Materials and Methods

### Plant materials and growth conditions

The near‐isogenic line *GS2™* (renamed by the previous *GS2* allele *GS2* ^*BDL*^) was obtained from the BC_9_F_2_ by repetitive backcrossing to *japonica* variety (*Oryza sativa* L. ssp. *japonica*) Zhonghua11 (ZH11) and 9311‐*GS2™* was developed from the BC_8_F_2_ by repetitive backcrossing to *indica* variety (*Oryza sativa* L. ssp. *indica*) 9311 (Hu *et al*., 2015). 9311‐*IPA1™* ^*1*^ was derived from BC_8_F_2_ by repetitive backcrossing *japonica* variety Shaoniejing to 9311 (Jiao *et al*., 2010) and WY‐*IPA1™* ^*2*^ (target site mutation2, TM2) was identified from *japonica* variety Wuye (WY) treated with EMS. WY‐*dep1* and 9311‐*dep1* were constructed from BC_8_F_2_ by the backcrossing *japonica* variety Wuyunjing 7 to WY and 9311, respectively. All plants were cultivated in the fields of the China National Rice Research Institute in Fuyang (Zhejiang province, 119°95′E, 30°05′N) and Lingshui (Hainan province, 110°02′E, 18°48′N) during the rice growing season. Each line was planted in eight rows and six plants per row, with a transplant spacing of 20 × 20 cm.

*GS2™* shows two base substitutions in the cleavage site of *OsmiR396c*, and both *IPA1™* ^*1*^ and *IPA1™* ^*2*^ display one base substitution in the target site of *OsmiR156* (Supporting Information Fig. S1a,b). These base variations in *GS2™*, *IPA1™* ^*1*^ and *IPA1™* ^*2*^ disrupt *OsmiR396c‐* or *OsmiR156*‐mediated transcript cleavages, leading to an increase in gene expression. The *dep1* allele contains a 625‐bp deletion, generating a premature stop codon (Fig. S1c). The genes’ genetic information is shown in the following Table 1.

**Table 1:** Genetic information of *GS2*, *GS2™*, *IPA1*, *IPA1™* ^*1*^, *IPA1™* ^*2*^, *DEP1* and *dep1*.

Genotype	Mutant form	Mutation site	Mutation result	Panicle phenotype
*GS2*	/	/	/	Normal
*GS2™*	TC → AA	487–488	Disrupted the cleavage of *OsmiR396c*	Increased grain size and panicle length
*IPA1*	/	/	/	Normal
*IPA1™* ^*1*^	C → A	874	Disrupted the cleavage of *OsmiR156*	Increased panicle branches, grain number and panicle length
*IPA1™* ^*2*^	C → T	876	Disrupted the cleavage of *OsmiR156*	Increased panicle branches, grain number and panicle length
*DEP1*	/	/	/	Normal
*dep1*	637‐bp deletion, 12‐bp insertion and premature stop	587–1211	Gain of function	Increased panicle branches and grain number, reduced grain size and panicle length

### Vector construction

To investigate the function of the *GS2* and *GS2™*, the knockout, RNAi and overexpression vectors were constructed. Gene knockout was carried out using the CRISPR‐Cas9 system, with *GS2* knockout vector transformed into both ZH11 and *GS2™* plants to generate homozygous knockout transgenic lines. The full‐length coding sequence (CDS) of *GS2™* was reverse complemented and inserted into RNAi vector p1300S. The *GS2™* RNAi vector was transformed into *GS2™* plants to generate *GS2™*‐Ri transgenic lines. Additionally, the CDS of *GS2* and *GS2™* were amplified and cloned into the *ProUbi* or *ProUbi‐GFP‐Flag* vector, which was then transformed into ZH11 plants to produce *GS2™*‐OE and *GS2*‐OE transgenic lines. All the positive transgenic plants have exceeded the T_8_ generation and exhibit stable inherited panicle morphology. In the T_0_ generation, we acquired more than six independent positive plants per transformation event, and finally, three homozygous lines were retained for continuous planting. Moreover, the CDS of *GS2* and precursor sequences of *OsmiR396c* were constructed into *ProUbi‐YFP‐HA* and *ProUbi‐GFP‐Flag*, respectively, and co‐transformed into protoplasts.

### Morphological and cellular analyses

The agronomic traits and yield per plant were measured after maturity. Grain numbers, grain sizes and 1000‐grain weight were calculated with an automated seed analyzer (Wan Shen SC‐E, Hangzhou, China), and grain thicknesses were measured by Vernier caliper. Young glume samples were fixed in 2.5% glutaraldehyde solution (30.5% 2 M Na_2_HPO_4_, 19.5% 2 M NaH_2_PO_4_ and 2.5% glutaraldehyde) at 4°C overnight, dehydrated with a graded ethanol series and critical drier (HCP‐2; Hitachi, Shanghai, China). Then, the samples were coated with platinum and examined with a scanning electron microscope (SU‐8010; Hitachi, Shanghai, China). Culms were fixed in FAA solution (formalin : glacial acetic acid : 70% ethanol; 1 : 1 : 14) overnight at 4°C following 15‐min vacuum treatment, dehydrated with a series of ethanol solutions (30%, 50%, 75%, 95%, 100%, 100% and 100%), cleared with a series of xylene solutions (50%, 70%, 90%, 100% and 100%) and embedded in paraplast (Sigma, Shanghai, China) for 2 d at 60°C. The sections were cut with a microtome (HM340E), stained with toluidine blue and examined by microscope (90I; Nikon, Tokyo, Japan).

### RT‐PCR and western blot

The plant RNA isolation kit (Axygen, Menlo Park, CA, USA) was used to extract total RNA from different organs. The reverse transcriptase kit (Toyobo, Shanghai, China) was used to synthesize complementary DNA from 2 μg of total RNA per sample. The reverse transcription polymerase chain reaction assay was carried out in triplicate with SYBR Green Master reagent, and rice *ACTIN* was used as the internal control for normalization. The primers used in this study are presented in Table S1.

The young panicles were ground in liquid nitrogen and then mixed with protein extraction buffer (62.5 mM Tris–HCl, pH 7.5, 2% SDS, 10% glycerol, 5% β‐mercaptoethanol and 1 mM PMSF). The mixture was vortexed thoroughly and incubated on ice for 20 min. After centrifugation at 1000 ***g*** for 5 min, the supernatant was supplemented with 5 × SDS loading buffer and denatured at 100°C for 5 min. Subsequently, the GS2‐specific peptide antibody was used for immunoblotting analysis to detect the expression level of GS2 protein. The antibody is based on a recombinant fusion protein containing a sequence corresponding to 120–394 amino acids of GS2/OsGRF4, which was reported in the reference of Song *et al*. (2023) and synthesized by Abclona Company (Palo Alto, CA, USA).

### Northern blot

At least 20 μg denatured RNA was loaded for 15% Urea‐PAGE electrophoresis and then transferred to the Nylon membrane (FFN15; Beyotime, Shanghai, China) by electrophoretic transfer; prehybridization was carried out for 2 h at 68°C. The probes anti‐U6 and anti‐OsmiR396c were labeled using Biotin 3′ End DNA Labeling Kit (D3106; Beyotime,), and hybridization was carried out at 68°C overnight. The biotin‐labeled probes were finally detected using the streptavidin‐HRP Kit (D3308; Beyotime).

### Subcellular localization

The cDNA sequence of *GS2* and *IPA1* was amplified and cloned into the C‐terminus of green fluorescent protein (GFP) driven by the 35S promoter. The *Pro35S::GFP*, *Pro35S::GS2‐GFP* and *Pro35S::IPA1‐GFP* vectors were transiently expressed in rice protoplasts and the fluorescence signals of GFP proteins were observed under confocal laser scanning microscopy (Leica TCS SP5) to reveal the subcellular locations of GS2 and IPA1. The primers used in this study are presented in Table S1.

### Yeast two‐hybrid assay

The yeast two‐hybrid (Y2H) screening library using 5–10 cm young panicles were established by Shanghai Ouyi Biomedical Technology Co. Ltd (Shanghai, China). Considering that GS2 exhibits self‐activation activity, the C‐terminal truncated protein of GS2 was used as the bait protein for Y2H to screen the possible interacting proteins (Fig. S2). The CDS of *GS2* and *IPA1* with different cDNA fragment were cloned into the pGBKT7 or pGADT7 vector (Clontech, Mountain View, CA, USA) and were transformed into the yeast strain Yeast Golden 2 with different combinations. The interactions were detected on SD/−Leu‐Trp medium and SD/−Leu‐Trp‐His‐Ade medium for growth, and pGBKT7‐53/pGADT7‐T and pGBKT7/pGADT7 were used as positive and negative control, respectively. The primers used in this study are listed in Table S1.

### Bimolecular fluorescence complementation assay

The cDNA of *GS2* from ZH11 and *GS2™* plants was cloned into pCAMBIA1300S‐YC vector, and *IPA1* was cloned into pCAMBIA1300S‐YN vector to obtain GS2‐YC, GS2™‐YC and IPA1‐YN, respectively. *Agrobacterium* strains transformed with *GS2*‐YC and IPA1‐YN or GS2™‐YC and IPA1‐YN constructs were collected and co‐infiltrated into leaves of *Nicotiana benthamiana*. The YN + GS2‐YC, YN + *GS2*™‐YC and IPA1‐YN + YC served as controls. Confocal microscopy (Zeiss LSM 710) was used to detect bimolecular fluorescence complementation (BIFC) fluorescent protein signals. The primers used in this study are listed in Table S1.

### Co‐immunoprecipitation assay

The CDS of *GS2* and *IPA1* were cloned into pCAMBIA1300S‐RFP‐HA and pCAMBIA1300S‐GFP‐Flag vectors to generate HA‐GS2 and Flag‐IPA1, respectively. The combined HA‐GS2 and Flag‐IPA1 vectors were co‐transformed into the rice protoplasts for 24 h. Total proteins were extracted with extraction buffer (150 mM NaCl, 50 mM Tris–HCl pH 7.5, 1 mM EDTA, 1% TritonX‐100, 1% glycerol and 1 mM PMSF) and incubated with Flag beads (M185‐11; MBL, Jiangsu, China) at 4°C for 2 h. The beads were washed three times with the wash buffer (150 mM NaCl, 50 mM Tris–HCl pH 7.5, 1 mM EDTA, 0.1% Triton X‐100 and 1 mM PMSF) and further boiled for 5 min with 50 μl 1 × SDS loading buffer and separated by 10% SDS‐PAGE. The Flag (M185‐7; MBL) and HA (M180‐7; MBL) antibodies were used to detect the immunoprecipitation, respectively. The primers used in this study are listed in Table S1.

### GST pull‐down assay

The full‐length CDS of *GS2* was inserted into the pET28a vector, and the His‐GS2 fusion protein was then induced by 1 mM IPTG at 18°C and subsequently purified by Ni‐NTA His Bind Resin (Millipore 70 666‐3). The full‐length CDS of *IPA1* was inserted into the pGEX‐4T‐1 vector and transformed into the *Escherichia coli* strain BL21 (DE3). Glutathione S‐transferase (GST)‐tagged proteins were induced by 1 mM IPTG at 18°C and bound by glutathione beads (A20022502; Smart‐Lifesciences, Changzhou, China). Approximately 0.5 μg of GST‐IPA1 purified protein was bound to GST beads at 4°C for 2 h; then, the same amount of His‐GS2 was incubated for 2 h and was finally collected by centrifugation and washed with pull‐down buffer (150 mM KCl; 50 mM Tris–HCL, pH 7.5; 1 mM EDTA; 1 mM DTT; 1 mM PMSF; 5% glycerol; and 0.01% NP‐40) five times. Finally, the output proteins were detected using His antibody (ab18184; Abcam, Shanghai, China) and GST antibody (ab92 Abcam). The primers used in this study are listed in Table S1.

### DAP‐seq assay

The full‐length CDS of *GS2* was inserted into the pDAP‐Halo‐Kan vector (Nanjing Zhongding Biotechnology Co., Ltd, Shangrao, China) and the Halo‐GS2 fusion protein was induced. Then, the fusion protein was incubated with the genomic DNA library and all DNAs bound to the transcription factor were isolated. Subsequently, high‐throughput sequencing was employed along with bioinformatics analysis to identify the binding sites of GS2. The analysis process included two biological replicates GS2‐1 and GS2‐2, as well as an input control for the DAP‐seq library. Illumina HiSeq was utilized to obtain raw data in PE150 bp fq format. Then, Bowtie2 (Langmead & Salzberg, 2012) was used to align the data to the genome, followed by peak calling using MACS (Zhang *et al.,* 2008), and idr software (Li *et al.,* 2011) was used to merge the biologically repeated peaks. Finally, the meme software (v.5.5.5) (https://meme‐suite.org/meme/tools/meme) was employed to analyze conserved motifs within the peak regions, while HOMER (http://homer.ucsd.edu/homer/) was used for peak annotation.

### Yeast one‐hybrid assay

The full‐length CDS of *GS2* was amplified from ZH11, and *GS2™* plants, and *IPA1* was cloned into the pB42AD vector to produce pB42AD‐*GS2*, pB42AD‐*GS2™* and pB42AD‐*IPA1*, respectively. The *DEP1*, *SRS3*, *LP*, *LG1* and *FZP* promoters were cloned into the pLacZ vector to generate pLacZ‐*DEP1*, pLacZ‐*SRS3*, pLacZ‐*LP*, pLacZ‐*LG1* and pLacZ‐*FZP* reporters. Then, the pB42AD‐*GS2*, pB42AD‐*GS2™* or pB42AD‐*IPA1* was co‐transformed with the reporter or control (empty pLacZ vector) into the yeast strain Yeast Golden 1 according to the Clontech transformation procedure. Transformants were cultured on SD/−Trp‐Ura plates containing β‐d‐galactopyranoside to check for possible interactions between GS2 or IPA1 and the reporter. The primers used in this study are listed in Table S1.

### Electrophoretic mobility shift assay

The GS2, GS2™ and IPA1 proteins were expressed and purified in *Escherichia coli* and electrophoretic mobility shift assay (EMSA) probes of *DEP1*, *SRS3*, *LP*, *LG1* and *FZP* promoters were commercially synthesized by Sunya Biological Technology (Hangzhou, China). The probes were labeled using an EMSA Probe Biotin Labeling Kit (GS008; Beyotime) and nonlabeled probes were used as competitors. The DNA‐binding reaction was performed for 20 min at 25°C and electrophoresed using 4% acrylamide gels. Then, the DNA probes were transferred to the Nylon membrane (FFN15; Beyotime), and oligo bands were finally detected using the streptavidin‐HRP Kit (GS009; Beyotime). The primers used in this study are listed in Table S1.

### Dual‐luciferase assay

Approximately 2‐kb promoter regions of *DEP1*, *SRS3*, *LP*, *LG1* and *FZP* were amplified and cloned into the upstream of luciferase (LUC) reporter gene to generate the *DEP1*‐LUC, *SRS3*‐LUC, *LP*‐LUC, *LG1*‐LUC and *FZP*‐LUC reporter constructs. The luciferase gene from *Renilla reniformis* (Ren) under the control of the CaMV35S promoter was used as the internal control. Full‐length cDNAs of *GS2*, *GS2™* and *IPA1* were amplified and inserted into the None vector to generate the d35S:*GS2*, d35S:*GS2™* and d35S:*IPA1* effector constructs, respectively. The combined reporter and effector plasmids were co‐transformed into the rice protoplasts. The LUC activity was quantified with a Dual‐Luciferase Assay Kit (E1910; Promega) following the product instructions, and relative LUC activity was calculated as the ratio of LUC/Ren. The primers used in this study are listed in Table S1.

### ChIP‐PCR assay

For the chromatin immunoprecipitation (ChIP)‐PCR assay, the chromatin was isolated from 2 g of young panicles of *GS2*‐overexpressing transplants according to a previous method (Wang *et al*., 2023). Immunoprecipitation was performed with protein G magnetic beads (78 608; Invitrogen) conjugated to GFP antibody (M185‐7; MBL), and HA antibody (M180‐7; MBL) was used as a negative control. The input samples were 2% volume of chromatin‐containing samples without immunoprecipitation treatment. The extracted DNA samples were used as template for reverse transcription polymerase chain reaction assay, and input samples were used as the control. The result was calculated according to the formula reported previously (Furlan‐Magaril *et al*., 2009). The primers used in this study are listed in Table S1.

### 5′ modified RACE

5′ modified rapid amplification of cDNA ends (RACE) was conducted according to a previous approach (Llave *et al*., 2002). Total RNA was extracted from the young panicle using TRIzol (Invitrogen). The primers GSP1 and GSP2 were used to perform the first and second PCRs, respectively. The products from the second PCR were purified by agarose gel electrophoresis, ligated into the pEASY‐Blunt Zero Cloning Vector (TransGen Biotech, Nanjing, China) and then sequenced. The primers used in this study are listed in Table S1.

## Results

### *GS2* negatively regulates grain number

**Fig. 1:**
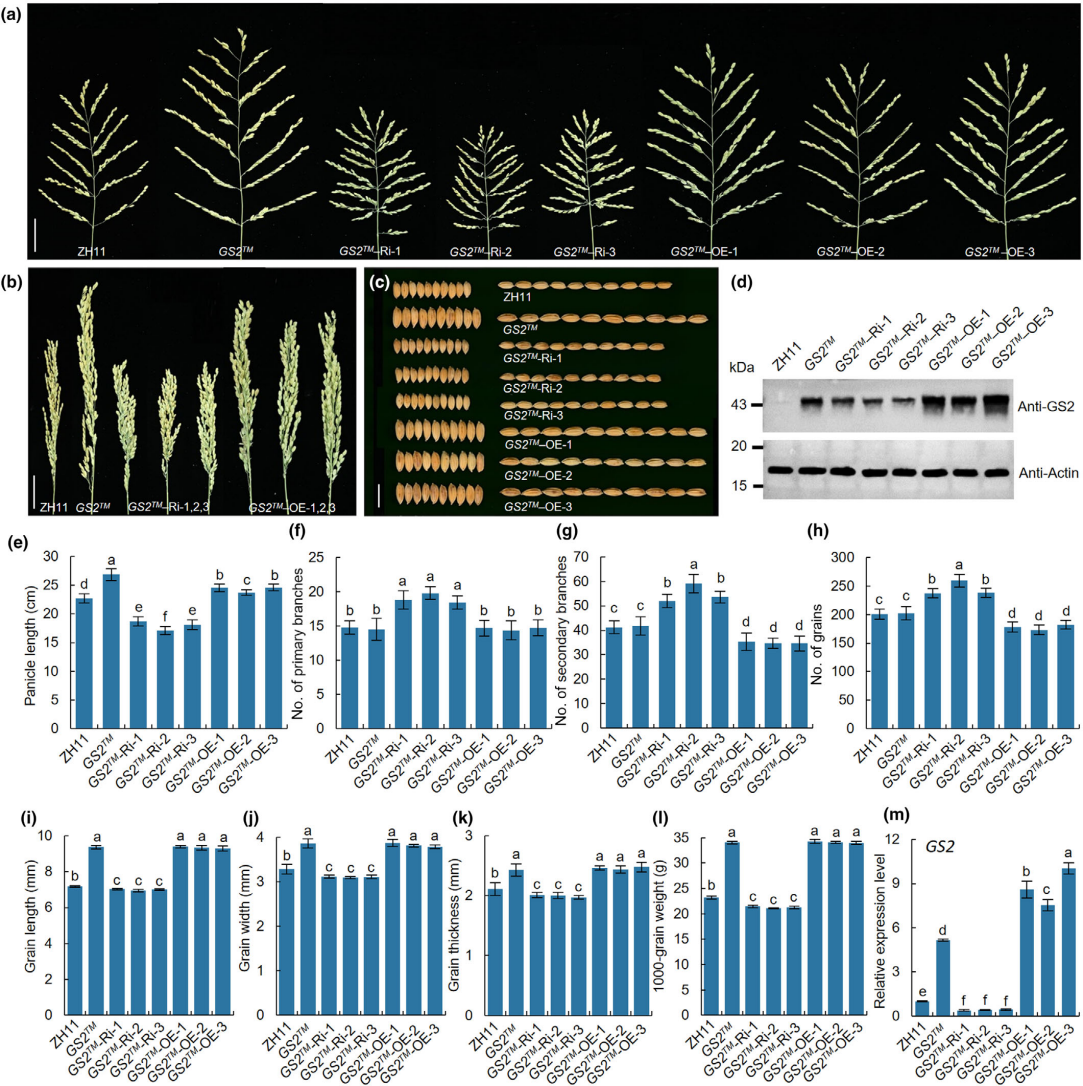
Phenotypic analysis of ZH11, *GS2™*, *GS2™*‐Ri and *GS2™*‐OE rice plants. (a, b) Panicle phenotypes of ZH11, *GS2™*, *GS2™*‐Ri and *GS2™*‐OE plants. Bars, 5 cm. (c) Grain phenotypes of ZH11, *GS2™*, *GS2™*‐Ri and *GS2™*‐OE plants. Bar, 1 cm. (d) The protein expression of GS2. (e–h) Panicle length, number of primary and secondary branches and number of grains in the main panicle (*n* = 20). (i–l) Grain length, width, thickness and 1000‐grain weight of ZH11, *GS2™*, *GS2™*‐Ri and *GS2™*‐OE grains (*n* = 20). (m) The RNA expression of *GS2*. RNA was extracted from the 5–10 cm young panicle of ZH11, *GS2™*, *GS2™*‐Ri and *GS2™*‐OE plants. Data are shown as means ± SD. One‐way ANOVA, letters indicate significant differences, *P* < 0.05.

To understand the underlying molecular mechanisms of *GS2* in panicle development, we carried out gene knockout and overexpression analysis (Fig. S3). The results showed that the all *GS2*‐KO and *GS2™*‐KO plants introduced premature stop codons due to different base insertions or deletions, resulting in loss of gene function (Figs S3c, S4d,e). There was no significant difference in phenotype between *GS2™*‐KO, *GS2*‐KO and wild‐type (WT) (Figs S3a,b, S4a,b,f), and the GS2 proteins were not detected in *GS2™*‐KO and *GS2*‐KO lines (Fig. S3d), suggesting that the loss‐of‐function mutation of *GS2* did not affect panicle and grain size development. However, all the *GS2™* overexpression lines can produce long panicles and super‐large grains (Fig. 1a–c,e–i), but only 4 of the 10 positive *GS2* overexpression lines increased *GS2* gene expression, which led to a slight increase in grain size (Fig. S4c,f,g,h), indicating that *GS2* may still be repressed or partially repressed by *OsmiR396c* in *GS2*‐OE plants. Considering that gene knockout directly disrupts the function of *GS2*, we used *OsmiR396c*‐resistant *GS2™* to construct RNAi expression vector and surprisingly found that transgenic plants showed an erect and dense panicle phenotype similar to *dep1* (Fig. 1a,b). The agronomic traits analysis revealed that *GS2™*‐Ri significantly increased the number of PBs, SBs and grains per panicle, but reduced plant height, panicle length and grain size, while *GS2™*‐OE decreased the numbers of SBs and grains per panicle, and increased panicle length and grain size (Figs 1a–c,e–l, S5–S7). Moreover, the reverse transcription polymerase chain reaction and western blot analysis showed that the RNA expression levels of GS2 were consistent with protein expression levels in *GS2™*‐Ri and *GS2™*‐OE lines (Fig. 1d,m). These results suggest that *GS2* negatively controls grain number and positively regulates grain size.

### *GS2™* disrupts the *OsmiR396c* ‐mediated transcript cleavage

**Fig. 2:**
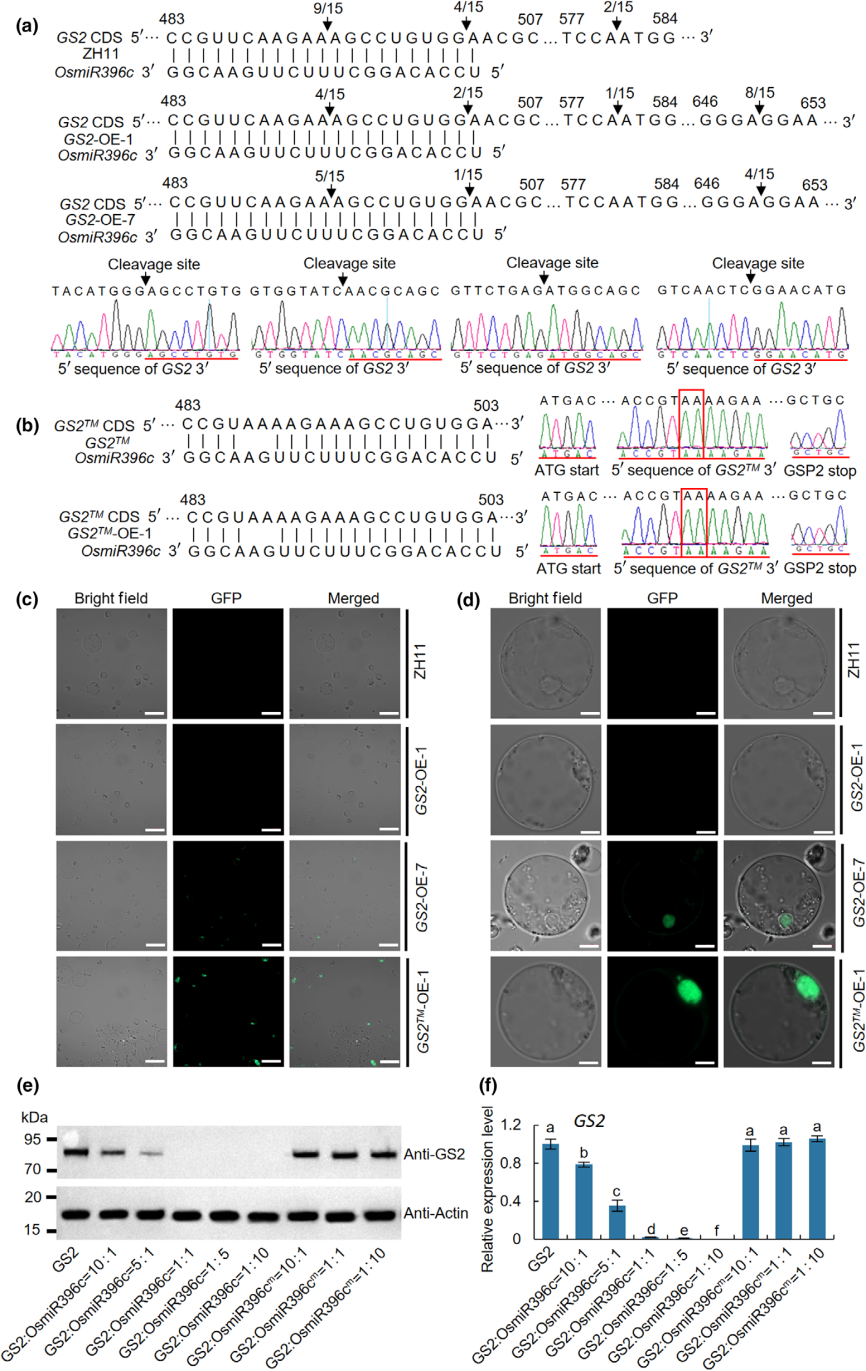
*OsmiR396c* cleavage rate in ZH11, *GS2*‐OE, *GS2™* and *GS2™*‐OE. (a) *OsmiR396c* cleavage sites in *GS2* mRNAs. The arrows represent the positions corresponding to the 5′ ends of the cleaved *GS2* mRNAs determined by 5′ rapid amplification of cDNA ends (RACE) and the frequency of 5′ RACE clones associated with each site. The arrowhead indicates the cleavage site identified by 5′ RACE. (b) *OsmiR396c* cleavage sites in *GS2™* mRNAs. Fifteen of 5′ RACE clones were sequenced. The red line represents the sequence that aligns with either *GS2* or *GS2™*. RNA was extracted from the young panicle of ZH11, *GS2*‐OE‐1, *GS2™* and *GS2™*‐OE‐1 plants. (c, d) Protoplasts were extracted from stems of ZH11, *GS2*‐OE and *GS2™* to analyze fluorescence signal, with (d) displaying magnified results of single cells from (c). Bars, 20 μm (c); 5 μm (d). (e) The protein expression levels of GS2 in rice protoplasts. (f) The RNA expression of *GS2*. RNA was extracted from rice protoplasts co‐transformed with *GS2* and *OsmiR396c*. Data are shown as means ± SD (*n* = 3). One‐way ANOVA, letters indicate significant differences, *P* < 0.05.

To further test the cleavage rate of *OsmiR396c*, we performed RLM‐RACE to detect *GS2* mRNA sequences in *GS2*‐OE and *GS2™*‐OE. As expected, the *GS2* mRNA was completely cleaved by *OsmiR396c* in ZH11 (*GS2* genotype) and was intact in *OsmiR396c*‐resistant *GS2™* and *GS2™*‐OE (Fig. 2a,b). Consistent with the RNA expression levels, *GS2* mRNA was partially cleaved by *OsmiR396c* in clones *GS2*‐OE‐7, 8, 9 and 10, whereas no intact sequence was found in clones *GS2*‐OE‐1, 2, 3, 4, 5 and 6, indicating that *GS2* may be genetically silenced or still within the repressive capacity of *OsmiR396c* in *GS2*‐OE‐1, 2, 3, 4, 5 and 6 (Figs 2a, S4c,f). Correspondingly, no fluorescence signals were detected in either ZH11 or *GS2*‐OE‐1 protoplasts, while strong and weak signals were observed in *GS2™*‐OE‐1 and *GS2*‐OE‐7 protoplasts, respectively (Fig. 2c,d). We further co‐expressed *GS2* and *OsmiR396c* in ZH11 protoplasts and found that GS2 protein was not detected when *OsmiR396c* was maintained at a high concentration (*GS2*: *OsmiR396c* = 1 : 1, *GS2*: *OsmiR396c* = 1 : 5 and *GS2*: *OsmiR396c* = 1 : 10) (Fig. 2e,f). Correspondingly, reverse transcription polymerase chain reaction and Northern blot analysis showed that the expression level of *OsmiR396c* was opposite to that of *GS2* (Fig. S8). These results revealed that *OsmiR396c* plays a strong repression role in the transcriptional regulation of *GS2*.

### GS2 physically interacts with IPA1

**Fig. 3:**
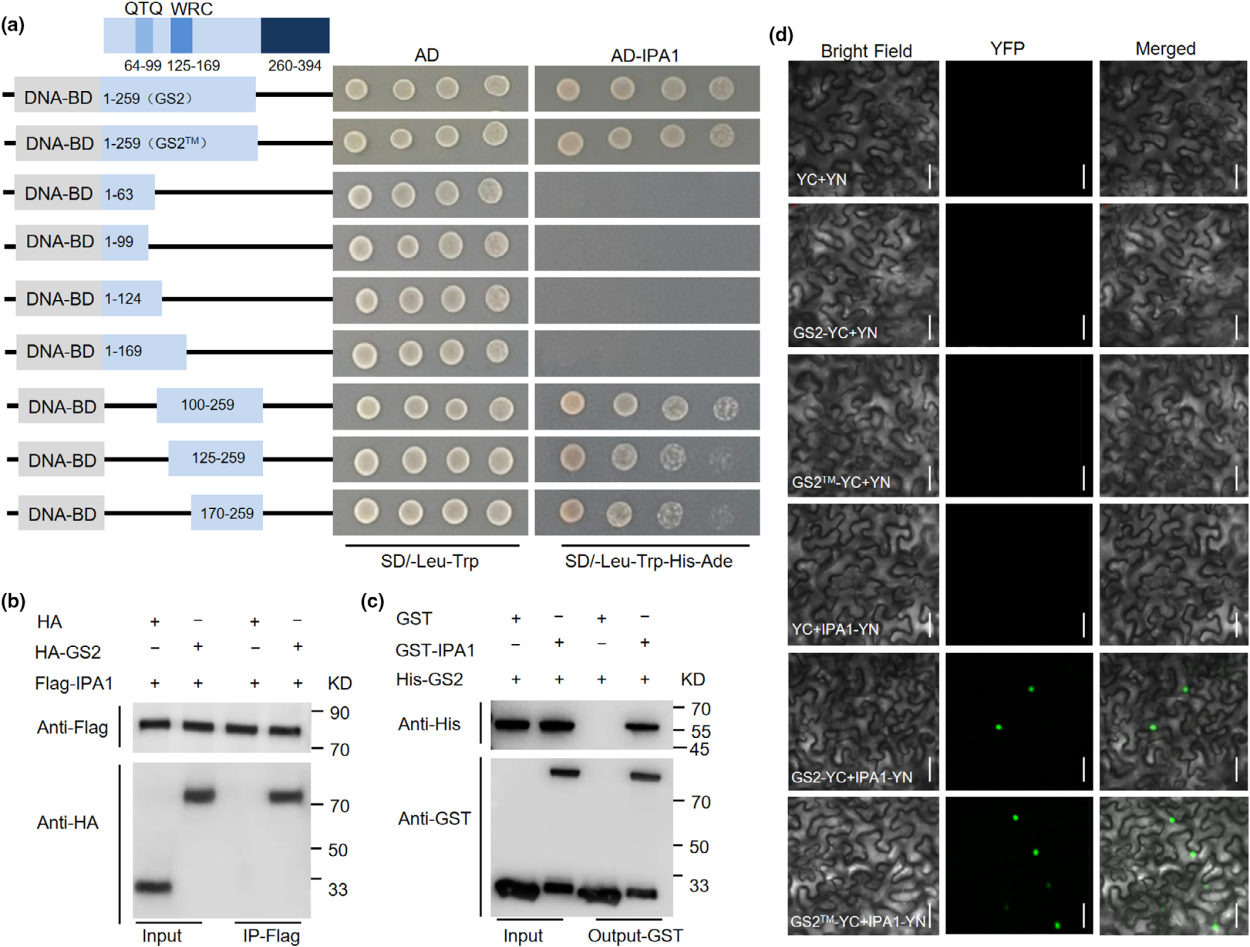
GS2 interacts with IPA1 protein. (a) Yeast two‐hybrid (Y2H) analysis of the interaction between GS2 and IPA1 proteins. The coding sequences of GS2 and GS2^TM^ with different cDNA fragment were fused to the DNA‐binding domain (BD), and IPA1 was fused to the transcription activation domain (AD). (b) Co‐immunoprecipitation (Co‐IP) assay showing that GS2 interacts with IPA1 proteins *in vivo*. Proteins were immunoprecipitated (IP) with Flag beads and analyzed by immunoblot (IB) using anti‐Flag and anti‐HA antibodies. A mixture of HA and Flag‐IPA1 was used as a negative control. (c) Glutathione S‐transferase (GST) pull‐down assay of the GS2 and IPA1 interaction. Anti‐His was used to detect the output protein. A mixture of GST and His‐GS2 was used as a negative control. (d) Bimolecular fluorescence complementation (BiFC) analysis of the interaction between GS2 and IPA1 proteins. The YFP fluorescence signals are pseudo‐colored as green. Bars, 20 μm. YC indicates YFP C‐terminal, YN indicates YFP N‐terminal.

To elucidate the underlying regulatory mechanism of *GS2* on panicle development, Y2H screening assay was conducted to identify the putative GS2‐interacting proteins. Finally, a total of six proteins were identified (Table S2), among which IPA1 was found to be involved in the differentiation of panicle branch. Subsequently, we constructed a set of truncations and deletions of GS2 and found that the C‐terminal region of 170–259 amino acids was sufficient to interact with IPA1 (Fig. 3a). To further verify the association of GS2 with IPA1, we performed a co‐immunoprecipitation assay *in vivo* and pull‐down assay *in vitro*. Two expression vectors *Pro35S::HA‐GS2* and *Pro35S::Flag*‐*IPA1* were transiently expressed in rice protoplasts, and the result showed that Flag‐IPA1 was successfully co‐immunoprecipitated with HA‐GS2 (Fig. 3b). Furthermore, the interaction between GS2 and IPA1 was further confirmed by the binding of GST fusion protein GST‐IPA1 to His‐GS2 in the pull‐down assay and the overlapping BIFC signals in the cell nucleus of *Nicotiana benthamiana* leaves (Fig. 3c,d). Moreover, Y2H vectors AD‐IPA1™^1^ and AD‐IPA1™^2^ containing the mutations in target site of *OsmiR156* were constructed (Fig. S9a,b) and the result of Y2H assay revealed that they also interacted with GS2 and GS2™ (Fig. S9c,d). Taken together, these results revealed that GS2 physically interacts with IPA1.

### *GS2™* and *IPA1™* enhance transcriptional activity

**Fig. 4:**
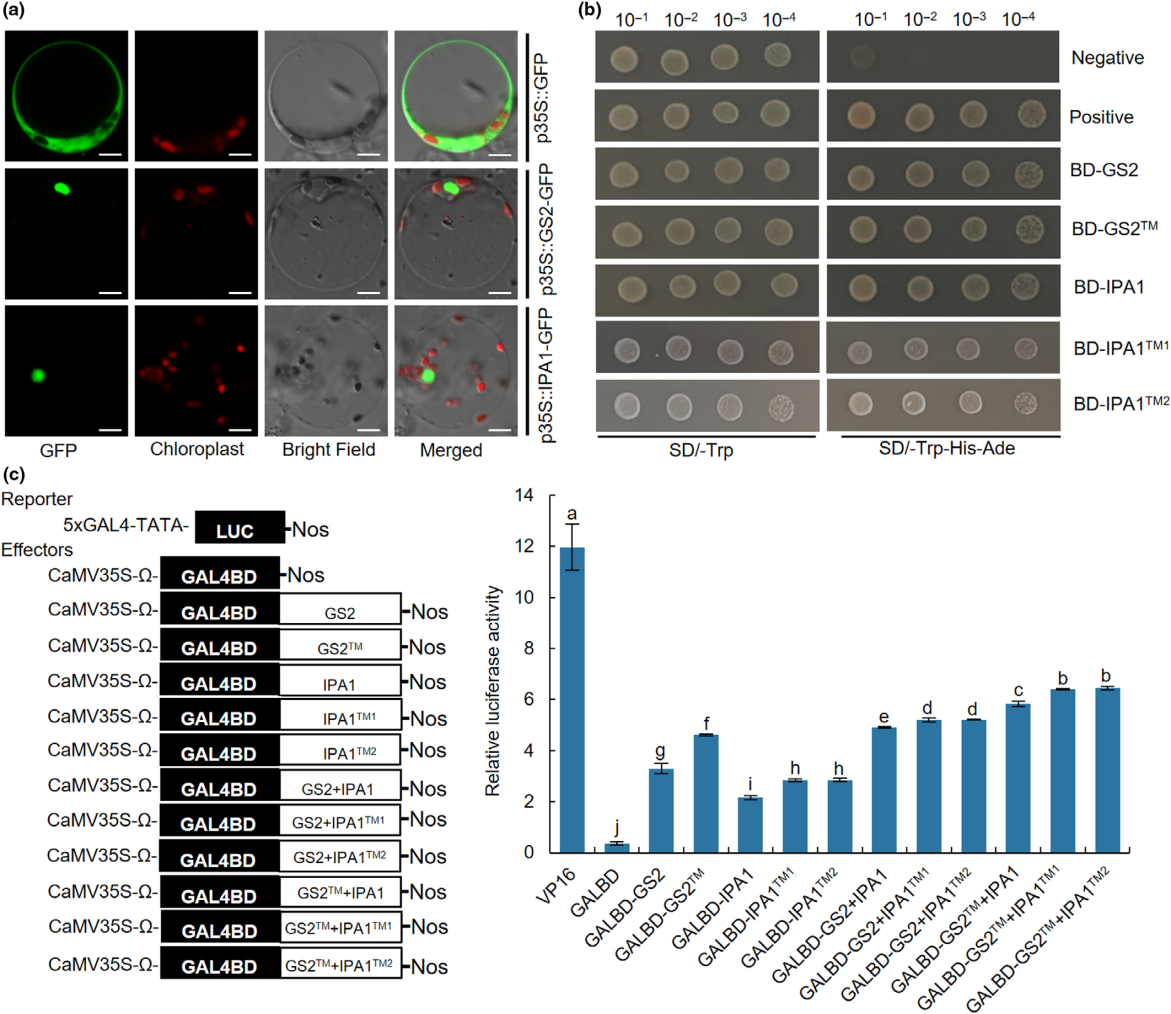
Transcriptional activity analysis of GS2 and IPA1 proteins. (a) GFP, GS2‐GFP and IPA1‐GFP fusion proteins in ZH11 protoplasts. Bars, 5 μm in all panels. (b) Yeast self‐activation assays for GS2, GS2^TM^, IPA1, IPA1^TM1^ and IPA1^TM2^. The negative control is an empty BD vector, while the positive control is the DST transcription factor, which demonstrates transcriptional activation activity in the yeast. (c) Relative luciferase activities of rice protoplasts (*n* = 4). Data are shown as means ± SD (*SD*). One‐way ANOVA, letters indicate significant differences, *P* < 0.05.

Transient expression of fusion proteins in rice protoplasts showed that GS2‐GFP and IPA1‐GFP were located in the nucleus (Fig. 4a), which were consistent with the function of GS2 and IPA1 as transcription regulators. To investigate the transcriptional activation ability of GS2™, IPA1™^1^ and IPA1™^2^, transcriptional activity analysis by yeast and LUC transient transcriptional assays were carried out. The result showed that all yeast cells fused with the full‐length cDNA of *GS2*, *GS2™*, *IPA1*, *IPA1™* ^*1*^ and *IPA1™* ^*2*^ to the GAL4 DNA‐binding domain, respectively, grew normally on SD/−Trp‐His‐Ade medium (Fig. 4b), indicating that GS2™, IPA1™^1^ and IPA1™^2^ also act as transcriptional activators. Moreover, the transcriptional activity of GS2™, IPA1™^1^ and IPA1™^2^ was significantly higher than that of GS2 and IPA1, respectively (Fig. 4c), revealing the higher potential ability of GS2™, IPA1™^1^ and IPA1™^2^ to promote target gene expression. Notably, the co‐transformation of GS2 + IPA1, GS2 + IPA1™^1^ and GS2 + IPA1™^2^ produced a stronger signal than single effector, and similar results were also observed in GS2™ + IPA1™^1^ and GS2™ + IPA1™^2^ (Fig. 4c). These results suggest that GS2™ and IPA1™^1^/IPA1™^2^ act as enhanced transcriptional activators and their co‐transformation can raise transcriptional activity.

### Screening of *GS2* target genes

**Fig. 5:**
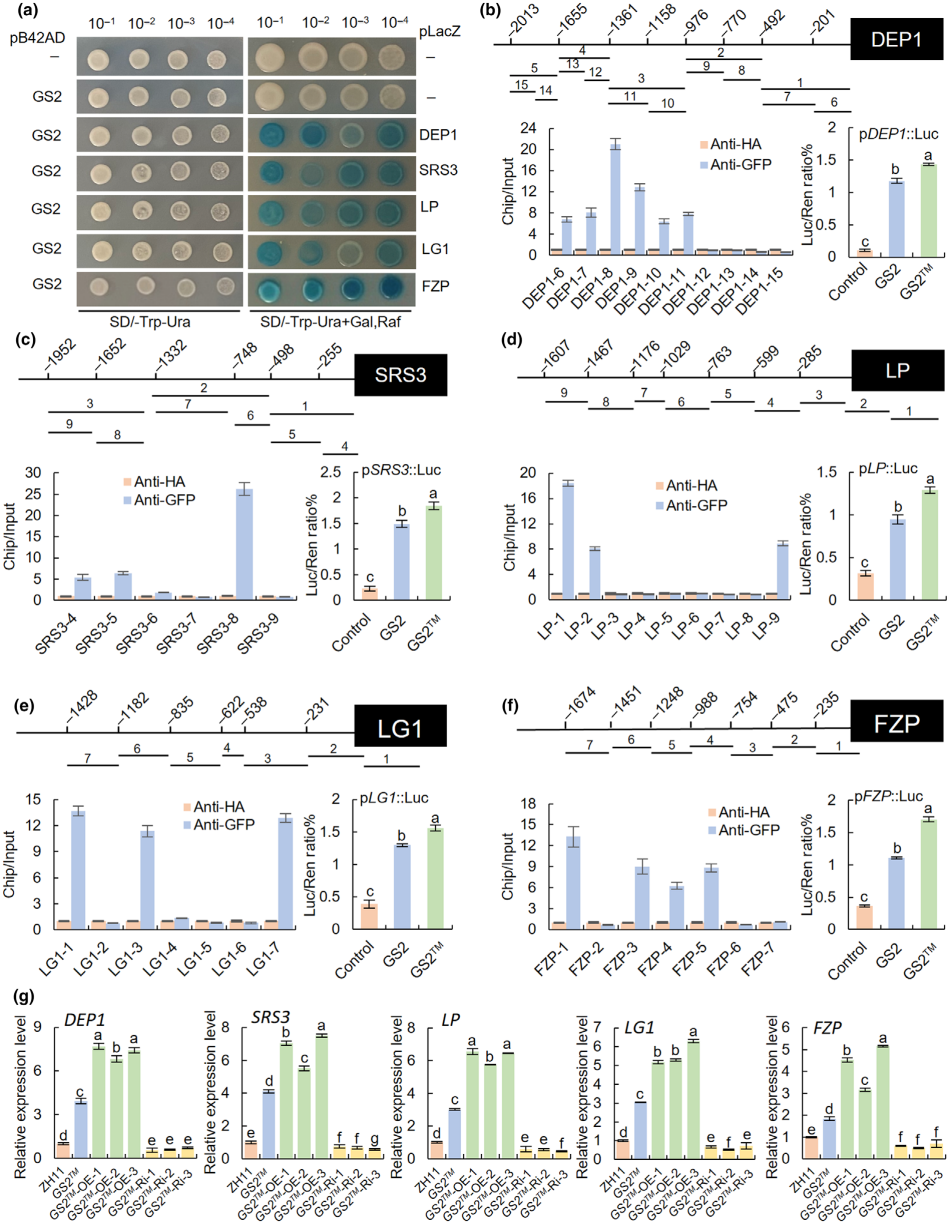
GS2 regulates the expression of *DEP1*, *SRS3*, *LP*, *LG1* and *FZP*. (a) Yeast one‐hybrid (Y1H) analysis of the interaction between GS2 and GS2™ with *DEP1*, *SRS3*, *LP*, *LG1* and *FZP*. The numbers above colonies represent the dilution gradients of the colonies. (b–f) Different fragments of the promoters of *DEP1*, *SRS3*, *LP*, *LG1* and *FZP*, and chromatin immunoprecipitation (ChIP)‐PCR and luciferase (LUC) transient transactivation assays showing that GS2 and GS2™ can significantly activate the transcription levels of *DEP1*, *SRS3*, *LP*, *LG1* and *FZP*. Data are shown as means ± SD (*n* = 4). (g) The RNA expression of *DEP1*, *SRS3*, *LP*, *LG1* and *FZP* in the young panicles of ZH11, *GS2™*, *GS2™*‐OE and *GS2™*‐Ri plants (*n* = 3). Data are shown as means ± SD. One‐way ANOVA, letters indicate significant differences, *P* < 0.05.

To understand the regulation pathway of *GS2* involved in panicle development, we systematically identified *GS2* target genes by DAP‐seq. The assay was performed using the genome DNA of ZH11, and the fragments specifically bound to GS2 protein were enriched and purified. Finally, a total of 1770 putative targets genes were identified (Table S3), including transcription factors, protein kinase and phosphorylase, DNA and RNA methyltransferase, ubiquitin ligase, Gγ subunit protein, microtubule associated protein, F‐box protein, DNA‐binding protein, cell cycle protein and auxin‐induced protein etc. Among them, *DEP1* (*LOC_Os09g26999*), *LP* (*LOC_Os02g15950*), *LG1* (*LOC_Os02g14730*), *SRS3* (*LOC_Os05g06280*) and *FZP* (*LOC_Os07g47330*) have been reported to be involved in panicle architecture and grain size (Table S3). We then conducted yeast one‐hybrid (Y1H) assays to verify these genes and found that the blue spots appeared in *DEP1*, *LP*, *LG1*, *SRS3* and *FZP* yeast cells, indicating that GS2 and GS2™ can activate LacZ reporter gene by binding their promoters (Figs 5a, S10a, S11a, S12a,b, S13a,b). Moreover, the promoters of *DEP1*, *LP*, *LG1*, *SRS3* and *FZP* were divided into 7 to 15 fragments, and ChIP‐PCRs were performed to detect the binding regions. The results showed that GS2 was significantly enriched on target regions of *DEP1* (DEP1‐6, DEP1‐7, DEP1‐8, DEP1‐9, DEP1‐10 and DEP1‐11), *LP* (LP‐1, LP‐2 and LP‐9), *LG1* (LG1‐1, LG1‐3 and LG1‐7), *SRS3* (SRS3‐4, SRS3‐5 and SRS3‐8) and *FZP* (FZP‐1, FZP‐3, FZP‐4 and FZP‐5) (Fig. 5b–f). These binding regions were also confirmed by yeast one‐hybrid assays (Figs S10a, S11a, S12a,b, S13a,b). Moreover, LUC transient transcriptional activity assay demonstrated that both GS2 and GS2™ activated *ProDEP1::LUC*, *ProLP::LUC*, *ProLG1::LUC*, *ProSRS3::LUC* and *ProFZP::LUC* reporter transcription (Fig. 5b–f). We also used reverse transcription polymerase chain reaction to detect the expression levels of *DEP1*, *LP*, *LG1*, *SRS3* and *FZP*, and found that these five genes were significantly upregulated in *GS2™*‐OE and significantly downregulated in *GS2™*‐Ri (Fig. 5g). These results supported that GS2 positively regulate the transcriptional expression of *DEP1*, *LP*, *LG1*, *SRS3* and *FZP*.

### GS2 and IPA1 co‐promote the expression of *DEP1*

**Fig. 6:**
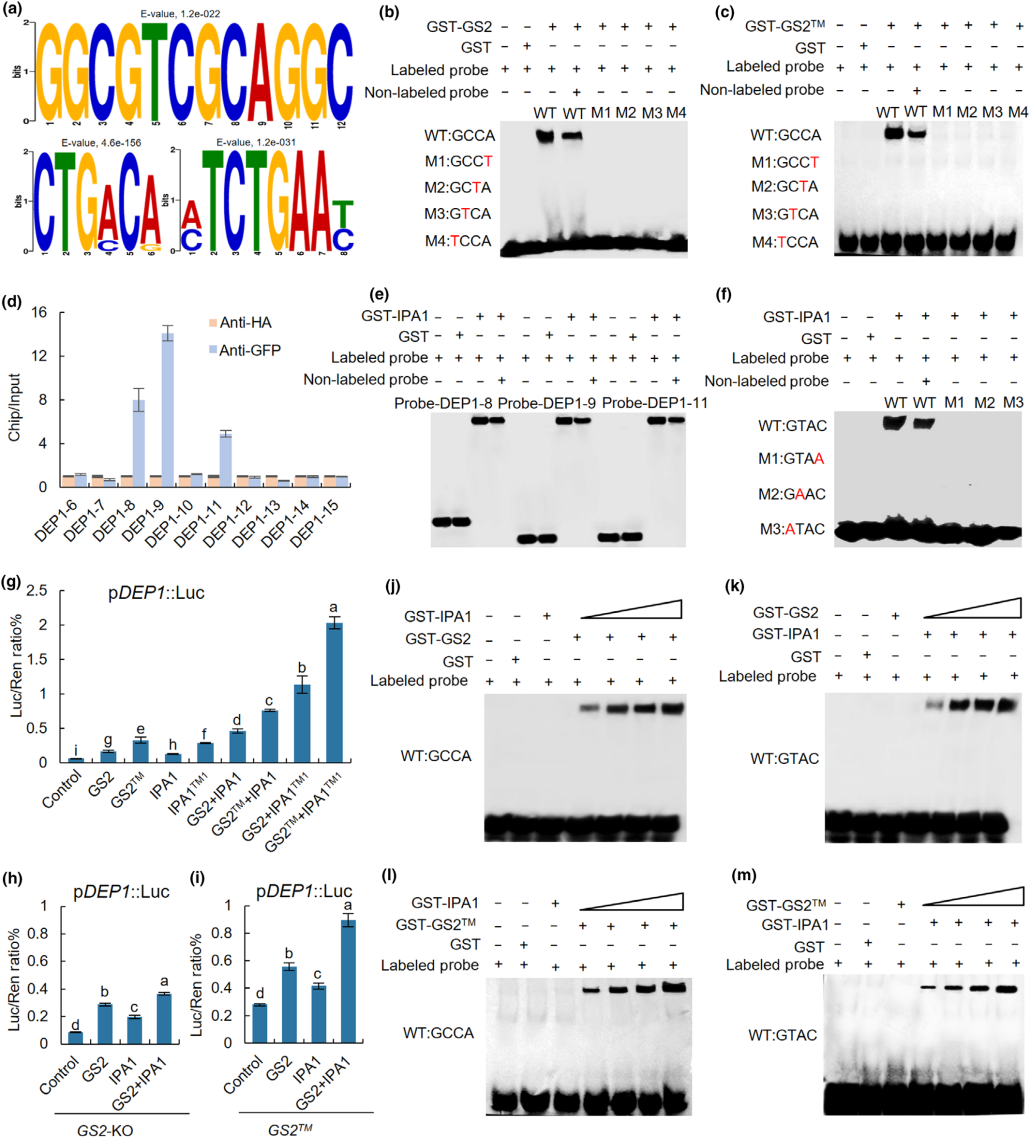
GS2 and IPA1 regulate the expression of *DEP1*. (a) Sequence motifs enriched in DAP‐seq with glutathione S‐transferase (GST)‐tagged GS2. (b, c) Electrophoretic mobility shift assay (EMSA) assays. GS2 and GS2™ can directly bind to the core sequence. (d) Chromatin immunoprecipitation (ChIP)‐PCR assay. IPA1 can directly bind to the DNA probes of *DEP1*. (e) EMSA assays. IPA1 can directly bind to the DNA probes of DEP1. (f) EMSA assays. IPA1 can directly bind to the core sequence. (g) Luciferase (LUC) transient transactivation assay in rice protoplasts. GS2, GS2™, IPA1 and IPA1™^1^ can significantly activate the transcription level of *DEP1*. (h, i) LUC transient transactivation assay in rice protoplasts in *GS2*‐KO (g) and *GS2™* (i). (j, l) EMSA assays. IPA1 proteins can enhance the binding of GS2 (j) and GS2™ (l) to the GCCA motif. (k, m) EMSA assays. GS2 (k) and GS2™ (m) proteins can enhance the binding of IPA1 to the GTAC motif. Data are shown as means ± SD (*SD*) (*n* = 4). One‐way ANOVA, letters indicate significant differences, *P* < 0.05.

Transcription factor binding site (TFBS) is a special DNA sequence, which is usually identified at the 5′ end of target gene and can be bound by transcription factors to regulate gene expression. Based on the DAP‐seq, three binding motifs of GS2 were identified (Fig. 6a). The first motif showed the conserved sequence GGCGTCGCAGGC, and the second and third motifs could be recognized by CTGA/CCA/G and A/CTCTGAAT/C, respectively (Fig. 6a). Electrophoretic mobility shift assays demonstrated that the binding DNA fragments of GST‐GS2 and GST‐GS2™ contained intact but not mutant GCCA core motif probes, and IPA1 could bind to GTAC motif probes (Fig. 6b,c,f). We further used EMSA to verify the binding specificity of GS2 and GS2™ to the enriched fragments of *DEP1*, *LP*, *LG1*, *SRS3* and *FZP*. The results revealed that the GST‐GS2 and GST‐GS2™ fusion proteins could bind to all these DNA probes containing GCCA motifs, and nonlabeled competing probes effectively reduced the binding ability of GST‐GS2 (Figs S10b, S11b, S12c,d, S13c,d). It has been reported that IPA1 can directly bind and activate *DEP1* to regulate panicle development (Lu *et al*., 2013). We carried out ChIP‐PCR and EMSA analysis of IPA1 to *DEP1* promoter (DEP1‐6, DEP1‐7, DEP1‐8, DEP1‐9, DEP1‐10, DEP1‐11, DEP1‐12, DEP1‐13, DEP1‐14 and DEP1‐15) and found that the regions of DEP1‐8, DEP1‐9 and DEP1‐11 were bound to IPA1 (Fig. 6d,e). Notably, GST‐IPA1 significantly enhanced the binding ability of GST‐GS2 to GCCA motif probes and GST‐GS2 also raised the binding ability of GST‐IPA1 to GTAC motif probes (Fig. 6j,k). Accordingly, GST‐GS2™ also showed an ability to bind GCCA motif probes, and the binding capacity to GCCA was elevated with the increase in GST‐IPA1 (Fig. 6l,m). Moreover, two factors (GS2 + IPA1, GS2™ + IPA1, GS2 + IPA1™^1^ and GS2™ + IPA1™^1^) displayed an obviously increased effect than single factor in activating *ProDEP1::LUC* reporter, among which the strongest was in GS2™ + IPA1™^1^ (Fig. 6g). We further extracted the protoplasts of *GS2*‐KO and *GS2*™ plants and found that the *ProDEP1::LUC* activity was still induced by GS2 or IPA1 and significantly enhanced when GS2 and IPA1 combined together (Fig. 6h,i). These results indicate that GS2 and IPA1 can mutually increase the binding ability to *DEP1* core motif.

### Double and triple mutants of *GS2™* , *IPA1™* ^*1*^ / *IPA1™* ^*2*^ and *dep1*

**Fig. 7:**
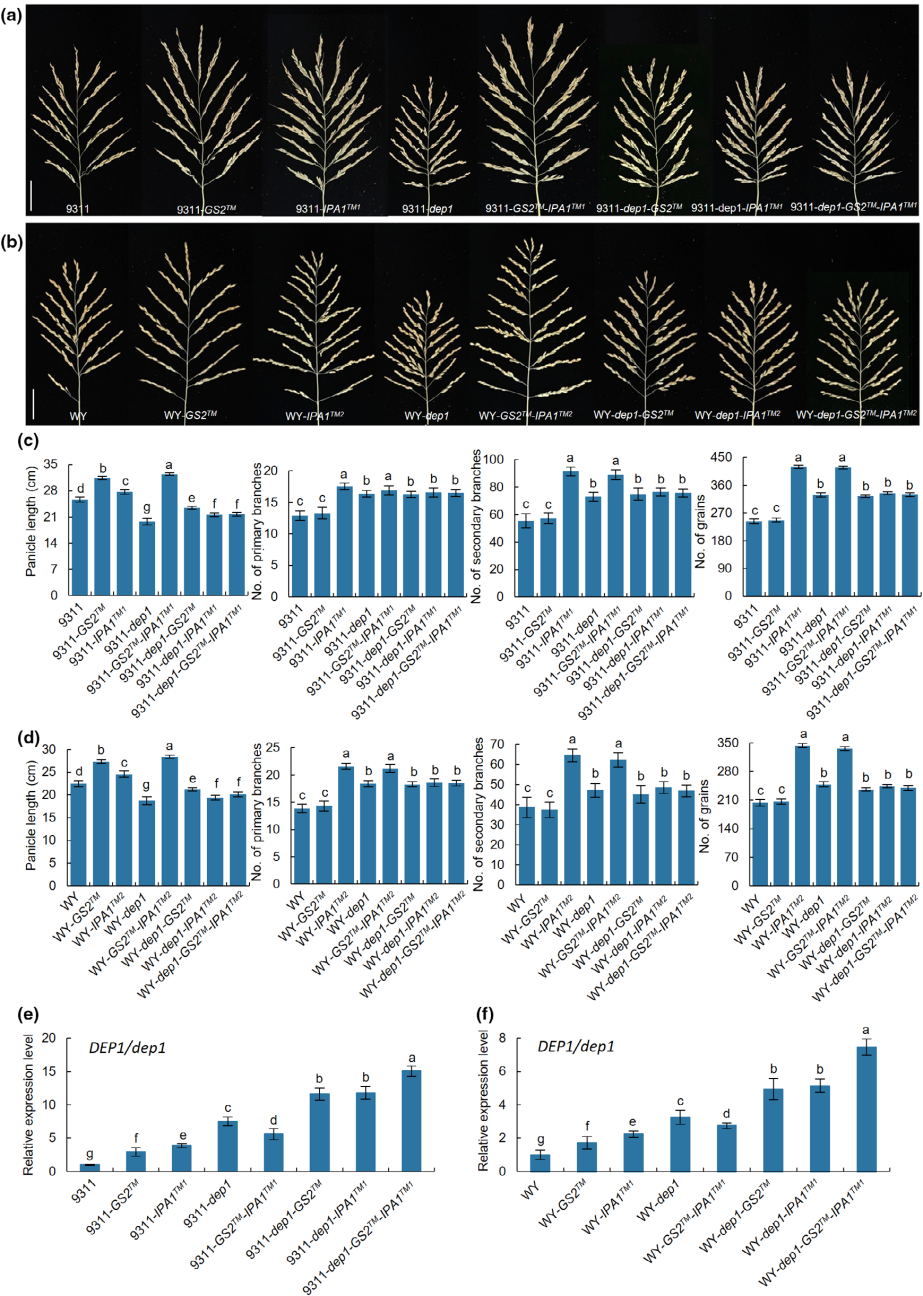
Genetic materials of *GS2*, *IPA1* and *DEP1*. (a, b) Panicle phenotypes of genetic materials of *GS2*, *IPA1* and *DEP1* in the background of 9311 (a) and WY (b) plants. Bars, 5 cm. (c, d) Panicle length, number of primary and secondary branches and number of grains in the main panicle (*n* = 20). (e, f) The RNA expression level of *DEP1*/*dep1*. RNA was extracted from the 5–10 cm young panicles. Data are shown as means ± SD (*SD*). One‐way ANOVA, letters indicate significant differences, *P* < 0.05.

To investigate the genetic relationship of *GS2*, *IPA1* and *DEP1*, we constructed different materials in the background of 9311 and WY and investigated the related agronomic traits (Figs 7a,b, S14). Compared with WT, *9311‐GS2™* and *WY‐GS2™* only increased panicle length, both *9311‐IPA1™* ^*1*^ and *WY‐IPA1™* ^*2*^ enhanced panicle length, panicle branches and grain number per panicle, while *9311‐dep1* and *WY‐dep1* decreased panicle length and raised panicle branches and grain number per panicle (Fig. 7c,d). Except for panicle length, the double mutants *9311‐GS2™‐IPA1™* ^*1*^ and *WY‐GS2™‐IPA1™* ^*2*^ showed the same number of panicle branches and grains per panicle as *9311‐IPA1™* ^*1*^ and *WY‐IPA1™* ^*2*^, but significantly more than *9311‐GS2™* and *WY‐GS2™*, indicating that *IPA1™* ^*1*^ and *IPA1™* ^*2*^ are epistatic to *GS2™* in panicle branches and grain number per panicle (Fig. 7c,d). Moreover, *9311‐dep1‐GS2™*, *WY‐dep1‐GS2™*, *9311‐dep1‐IPA1™* ^*1*^ and *WY‐dep1‐IPA1™* ^*2*^ also exhibited significantly raised PBs, SBs and grain number per panicle than those of WT, but there was no significant difference with *dep1* (Fig. 7c,d). Similar phenotypes were also found in triple mutants *9311‐dep1‐GS2™‐IPA1™* ^*1*^ and *WY‐dep1‐GS2™‐IPA1™* ^*2*^, suggesting that *dep1* is epistatic to *GS2™*, *IPA1™* ^*1*^ and *IPA1™* ^*2*^ in panicle branches and grain number per panicle (Fig. 7c,d). We further detected RNA expression levels and found that *DEP1*/*dep1* was highly expressed in all test materials except WT (Fig. 7e,f). The expression level of *DEP1*/*dep1* was enhanced in *9311‐GS2™* and *9311‐IPA1™* ^*1*^ and also significantly increased in double and triple mutants *9311‐dep1‐GS2™*, *9311‐dep1‐IPA1™* ^*1*^ and *9311‐dep1‐GS2™*‐*IPA1™* ^*1*^, indicating that *GS2™*, *IPA1™* ^*1*^ and *IPA1™* ^*2*^ can significantly increase the expression of *DEP1*/*dep1*. These findings reveal that *GS2* and *IPA1* act upstream of *DEP1* and synergistically regulates the expression of *DEP1* to control panicle branches and grain number per panicle.

### *GS2™* and *IPA1™* ^*1*^ promotes yield increase in hybrid rice

**Fig. 8:**
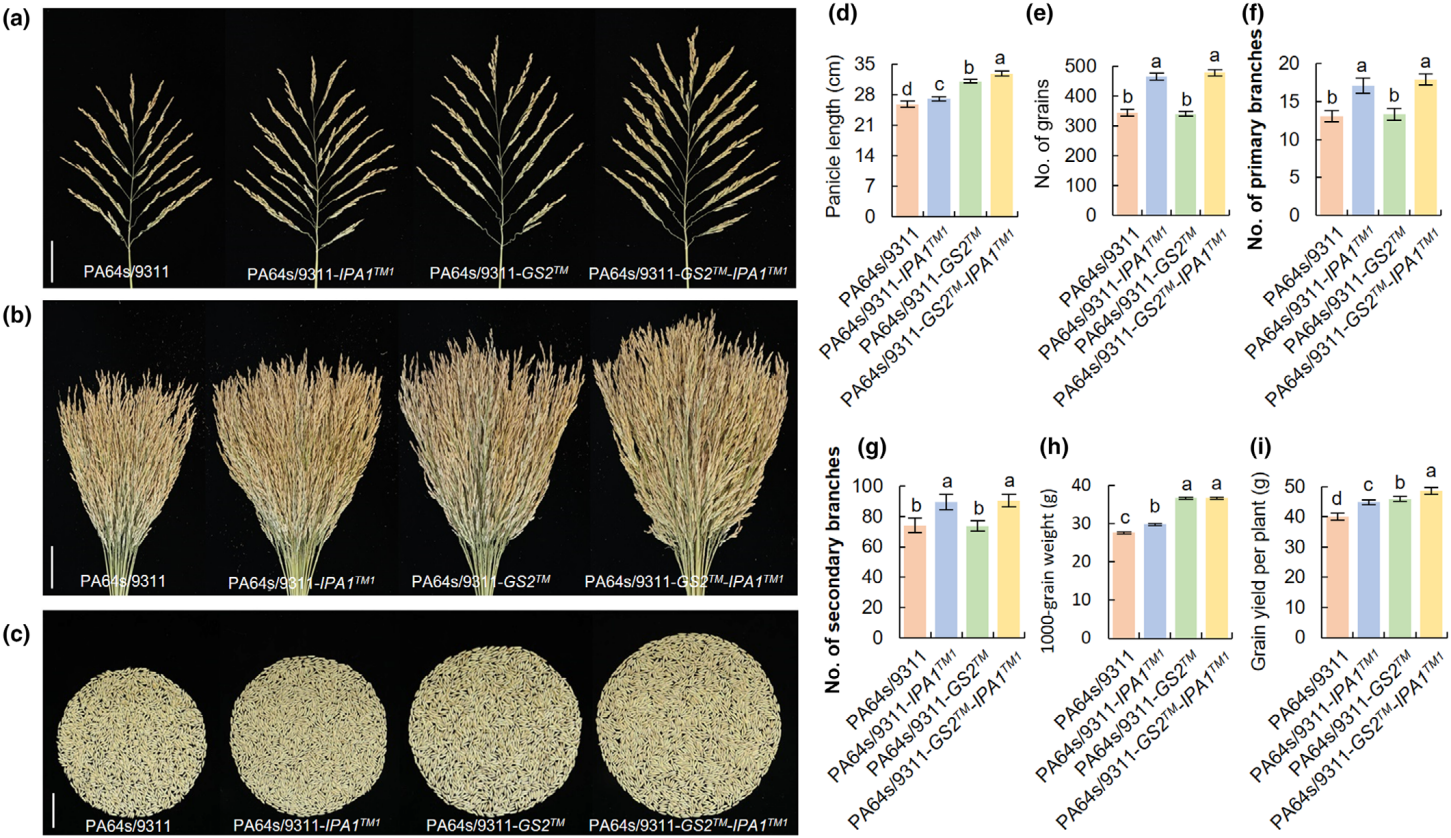
Pyramiding of *GS2™* and *IPA1™* ^*1*^ on hybrid rice. (a, b) Comparison of panicles of PA64s/9311, PA64s/9311‐*IPAI™* ^*1*^, PA64s/9311‐*GS2™* and PA64s/9311‐*GS2™*‐*IPA1™* ^*1*^. Bars, 5 cm. The number of 25 panicles is shown in (b). (c) Comparison of grains per plant in PA64s/9311, PA64s/9311‐*IPAI™* ^*1*^, PA64s/9311‐*GS2™* and PA64s/9311‐*GS2™*‐*IPA1™*. Bar, 5 cm. (d–g) Panicle phenotypes of PA64s/9311, PA64s/9311‐*IPAI™* ^*1*^, PA64s/9311‐*GS2™* and PA64s/9311‐*GS2™*‐*IPA1™* (*n* = 10). (h) 1000‐grain weight (*n* = 20). (i) Grain yield per plant (*n* = 20). Data are shown as means ± SD. One‐way ANOVA, letters indicate significant differences, *P* < 0.05.

Considering that heterozygous *GS2* and *IPA1* are more suitable for production (Jiao *et al*., 2010; Hu *et al*., 2015), we used two‐line male sterile line PA64s to breed a series of hybrid rice combinations. The grain yield and other agronomic traits were evaluated under field conditions. As shown in Fig. 8a,b,d, the panicle length was increased gradually in the order of PA64s/9311, PA64s/9311‐*IPA1™* ^*1*^, PA64s/9311‐*GS2™* and PA64s/9311‐*GS2™*‐*IPA1™* ^*1*^. The PBs, SBs and grain number per panicle of PA64s/9311 and PA64s/9311‐*GS2™* were significantly less than those of PA64s/9311‐*IPA1™* ^*1*^ and PA64s/9311‐*GS2™*‐*IPA1™* ^*1*^ (Fig. 8e–g). However, the 1000‐grain weights of PA64s/9311‐*GS2™* and PA64s/9311‐*GS2™*‐*IPA1™* ^*1*^ were significantly higher than that of PA64s/9311 and PA64s/9311‐*IPA1™* ^*1*^ (Fig. 8h). Under the effect of increasing 1000‐grain weight and grain number per panicle, PA64s/9311‐*GS2™*‐*IPA1™* ^*1*^ showed an increase of 21.32% in grain yield per plant, while PA64s/9311‐*IPA1™* ^*1*^ and PA64s/9311‐*GS2™* increased by 12.14% and 13.95%, respectively (Fig. 8b,c,i). Our result revealed that the pyramiding of *GS2™* and *IPA1™* ^*1*^ has great potential in breeding super‐high‐yield rice.

## Discussion

### *GS2™* is an important determinant of panicle architecture

Growth‐regulating factors (GRFs) are plant‐specific transcription factors, which are defined by the presence of two highly conserved protein domains WRC and QLQ (Omidbakhshfard *et al*., 2015). In rice, *OsGRFs* consists of 12 members (*OsGRF1*‐*OsGRF12*) and are mainly involved in the growth regulation of plant architecture, leaf size, tiller number, heading date, floral organogenesis and grain size (Luo *et al*., 2005; Kuijt *et al*., 2014; Liu *et al*., 2014; Hu *et al*., 2015; Kim & Tsukaya, 2015; Chen *et al*., 2020). It has been reported that *OsGRF4*, *OsGRF6* and *OsGRF8* are directly regulated by *OsmiR396* (Hu *et al*., 2015; Tang *et al*., 2018; Dai *et al*., 2019). Among them, *GS2* encodes an OsGRF4 transcription factor which plays a positive regulatory role in grain size (Hu *et al*., 2015). The two bases substitution TC to AA in *GS2* target site disrupts *OsmiR396c*‐mediated transcript cleavage, resulting in a significant increase in grain size and grain yield (Che *et al*., 2015; Duan *et al*., 2015; Hu *et al*., 2015). Except for a new gain‐of‐function allele *GS2* ^E^ generated by CRISPR/Cas9 (Wang *et al*., 2022), all reported *GS2* genes identified from different rice varieties, including *GRF4*, *GS2* ^*BDL*^, *GS2* ^*AA*^, *GL2*, *LGS1*, *GLW2* and *PT2*, displayed the same TC to AA mutation sites and similarly increased grain size and yield, indicating that these varieties may all come from the breeding improvement of an original parent (Che *et al*., 2015; Duan *et al*., 2015; Hu *et al*., 2015; Sun *et al*., 2016; Chen *et al*., 2019). However, no significant panicle phenotypic changes (panicle length, number of PBs and SBs and number of grains) were observed and no GS2 protein was detected in *GS2™*‐KO and *GS2*‐KO lines (Figs S3, S4). It is well understood that the *GS2™*‐KO and *GS2*‐KO caused the premature termination of GS2 protein translation, leading to the loss of GS2 function (Fig. S3c,d), which is similar to that *OsmiR396c*‐mediated cleavage inhibits the expression of GS2 protein in WT (Figs 2a, S3d). Furthermore, we detected the *OsmiR396c*‐directed cleavage sequences and expression levels of *GS2* in *GS2*‐OE lines. No complete *GS2* fragment was found in *GS2*‐OE‐1, 2, 3, 4, 5 and 6 clones, but 5/15 complete *GS2* forms were detected in *GS2*‐OE‐7 clone (Fig. 2a), indicating that the *OsmiR396c* could not completely repress the expression of *GS2* in *GS2*‐OE‐7. Similar scenario was also found in the target gene *NAC2* of *OsmiR164b*. The Os*miR164b*‐resistant *OsNAC2* (OErN) prevents *OsmiR164b*‐mediated transcript cleavage, and its overexpression exhibited longer panicles and more grains, while no phenotypic and agronomic differences were discovered in *OsNAC2‐*overexpressing plants (Jiang *et al*., 2018), which may be due to the *OsmiR164b* play a strong repression on the target gene *OsNAC2*. To avoid the influence of *OsmiR396c* to *GS2*, the *OsmiR396c*‐resistance *GS2™*‐Ri and *GS2™*‐OE were conducted. As expected, *GS2™*‐Ri and *GS2™*‐OE showed a significant reduced and increased grain size, respectively, which is consistent with previous reports that *GS2* plays a positive role in controlling grain size (Hu *et al*., 2015; Chen *et al*., 2019). Moreover, *GS2™*‐Ri also produced short dense panicle with increased PBs and SBs and grain number per panicle (Fig. 1a,b,e–h). On the contrary, *GS2™*‐OE showed longer panicles and fewer grains than WT (Fig. 1a,b,d,e–h). Therefore, *GS2™* acts as a positive regulator in controlling panicle length and grain size, and as a negative regulator in regulating panicle branches and grain number.

### The interaction between GS2 and IPAl promotes the expression of downstream effectors

By screening rice yeast library, the interacting protein IPA1 of GS2 was identified and verified (Fig. 3a). *IPA1* is a functional transcriptional activator that plays an important role in regulating plant and panicle architecture (Jiao *et al*., 2010). Previous reports have revealed that IPA1 directly positively regulates *DEP1*, affecting plant height and panicle length (Lu *et al*., 2013). Interestingly, GS2 can also bind to the GCCA motif of *DEP1* promoter region and positively regulates its expression (Fig. 6a–c). Moreover, *GS2™*‐Ri produced an erect dense panicle with increased panicle branches and grain number similar to *dep1* (Fig. 1a,b,e–h). Combined with the phenotypes of *dep1* in panicle branch and grain number per panicle is epistatic to *GS2* and *IPA1* (Fig. 7a–d), we speculated that *DEP1* may be a common downstream target gene of IPA1 and GS2. However, the panicle lengths of double mutants *GS2™‐IPA1™* ^*1*^ and *GS2™‐IPA1™* ^*2*^ showed an additive effect, and were longer than those of *dep1* in the two backgrounds of 9311 and WY, respectively (Fig. 7a–d), suggesting that *GS2* and *IPA1* may regulate panicle length independently of *dep1*. Previous reports have shown that multiple target genes of *GS2* were identified by ChIP‐seq (Li *et al*., 2018). Among them, only 19 genes overlapped, such as *DEP1* and *LP* in ChIP‐seq and DAP‐seq results (Table S4), which may be due to the difference that DAP‐seq enriched genes derived from young panicles, while ChIP‐seq from seedlings under high nitrogen conditions. It has been reported that *dep1* is an important natural variation regulating panicle architecture, which improves grain yield and NUE (nitrogen use efficiency) by increasing N and dry matter transport (Huang *et al*., 2009; Sun *et al*., 2014; Huang *et al*., 2022). Another target gene *LP* encodes an F‐box protein that interacts with SKP1‐like protein and may be involved in repressing cytokinin level to enhance plant height, panicle branches and grain numbers (Li *et al*., 2011; Yu *et al*., 2015). It has been reported that some protein interactions can enhance the expression of downstream target genes by transactivation (Wang *et al*., 2023). *GR5* encodes an AP2 transcription factor, whose interaction with Gγ subunits DEP1 and RGG2 can promote GR5 to elevate the expression of target genes (Wang *et al*., 2023). MADS transcription factor qLGY3/OsLG3b/MADS1 interacts with GS3 and DEP1 to enhance OsMADS1 transcriptional activity and promotes the co‐operative transactivation of target genes (Liu *et al*., 2018). *PAL1* is a CK receptor OHK4/OsHK4, which is positively regulated by IPA1 based on the interactions of IPA1 with PCF1 and PCF2 (Chun *et al*., 2023). Similarity, the interaction between GS2 and IPA1 increased their binding ability to *DEP1*, resulting in a significant increase in *DEP1* expression (Figs 6g–i, 7e,f). Moreover, the interaction also promoted GS2 to bind *LP*, *LG1*, *SRS3* and *FZP*, and significantly enhanced their expression (Figs 5g, S15). Especially, the *GS2™* disrupted *OsmiR396c*‐mediated transcript cleavage, and *IPA1™* ^*1*^ and *IPA2™* ^*2*^ interfered with *OsmiR156*‐mediated transcript cleavage, resulting in higher expression of their downstream genes than *GS2* and *IPA1*, respectively (Figs 4c, 7e,f, S15).

### The pyramiding of *GS2™* and *IPA1™* ^*1*^ on hybrid rice increased rice yield

Improving rice yield potential is always the main goal of breeding program in many countries. At present, gene pyramiding has been widely considered as an effective strategy to improve target traits. To breed high‐yielding varieties, the elite genetic gene resources are essential. It has been reported that *GS2* and *IPA1* are yield‐increasing genes and their dominant alleles display different yield effects (Jiao *et al*., 2010; Hu *et al*., 2015). Recent reports have indicated that only one parent needs improvement for the dominant (partial dominant) effect loci, while the additive effect or negative dominant effect loci must be improved by both parents to maximize the heterosis in the breeding of hybrid rice parents (Gu *et al*., 2023). Fortunately, both *GS2™* and *IPA1™* ^*1*^ are dominant effectors, which can provide great convenient for their application in hybrid rice. However, homozygous dominant *GS2* will reduce appearance quality due to the increase in grain size, while homozygous dominant *IPA1* will affect the yield potential due to the decrease in tiller number (Jiao *et al*., 2010; Hu *et al*., 2015; Zhang *et al*., 2017). Therefore, their heterozygous state is more suitable for achieving optimal yield and quality requirements in rice production. The super‐hybrid rice LYP9 (PA64s/9311) is one of the most popular varieties in China, which is derived from the cross between male parent 9311 and female parent PA64s (Zuo & Li, 2014). We constructed near‐isogenic lines 9311‐*GS2™*, 9311‐*IPA1™* ^*1*^ and gene pyramiding materials 9311‐*GS2™*‐*IPA1™* ^*1*^ through backcrossing with 9311 (Fig. 7a,c) and found that the grain yield of hybrid combination PA64s/9311‐*GS2™*‐*IPA1™* ^*1*^ was significantly higher than that of PA64s/9311, PA64s/9311‐*GS2™* and PA64s/9311‐*IPA1™* ^*1*^ (Fig. 8i). Statistical data showed that the yield‐enhancing effect was mainly attributed to the increase in 1000‐grain weight by *GS2™* and grain number by *IPA1™* ^*1*^ (Fig. 8e–h). Our results revealed that the pyramiding of *GS2™* and *IPA1™* ^*1*^ can significantly increase rice yield, which provides an excellent material for super‐hybrid rice breeding.

## Competing interests

None declared.

## Author contributions

Yuexing Wang, QQ and JH designed the research. Yueying Wang, JW and PH prepared materials. KW, BC, JL, Y Wu, LZ, ND, YT and HW performed phenotypic evaluations. GZ, LZ, DR and QZ provided technical support. JH, Yueying Wang and YL performed analysis and interpretation of the data. JH, Yueying Wang and YL drafted the manuscript. Yuexing Wang and SG contributed ideas for the article. All authors have read and agreed to the published version of the manuscript. Yueying Wang, YL and Y Wen contributed equally to this work.

## Disclaimer

The New Phytologist Foundation remains neutral with regard to jurisdictional claims in maps and in any institutional affiliations.

## Supporting information


**Fig. S1** Different mutation forms of *GS2*, *IPA1* and *DEP1*.
**Fig. S2** The self‐activation activity of GS2 and GS2™ proteins with 1–259 amino acids in yeast cells.
**Fig. S3** The effect of GS2 on phenotypes in ZH11, *GS2™*, *GS2™*‐KO and *GS2*‐KO plants.
**Fig. S4** Phenotype analysis of ZH11, *GS2™*, *GS2™*–KO, *GS2*‐KO and *GS2*‐OE plants.
**Fig. S5** Analysis of the cell size of ZH11, *GS2™*, *GS2™*–Ri and *GS2™*–OE grains.
**Fig. S6** The effect of the *GS2* on phenotypes of ZH11, *GS2™*, *GS2™*–Ri and *GS2™*–OE plants.
**Fig. S7** Stem traits of *GS2™* and *GS2™*‐Ri.
**Fig. S8** The expression of *OsmiR396c*.
**Fig. S9** The Interactions between GS2, GS2™ and IPA1™^1^, IPA1™^2^ proteins.
**Fig. S10** GS2 regulates the expression of *DEP1*.
**Fig. S11** GS2™ regulates the expression of *DEP1*.
**Fig. S12** *GS2* regulates the expression of *SRS3* and *LP*.
**Fig. S13** *GS2* regulates the expression of *LG1* and *FZP*.
**Fig. S14** The effect of the *GS2*, *IPA1* and *DEP1* on phenotypes of 9311 and WY plants.
**Fig. S15** The expression of *FZP*, *LG1*, *LP* and *SRS3*.

**Table S1** Primers used in this study.
**Table S2** The result of the interaction proteins with GS2.
**Table S3** The result of DAP‐Seq of GS2.
**Table S4** The common genes in ChIP‐seq and DAP‐seq results of GS2/OsGRF4.
Please note: Wiley is not responsible for the content or functionality of any Supporting Information supplied by the authors. Any queries (other than missing material) should be directed to the *New Phytologist* Central Office.

## Data Availability

All data related to this manuscript can be found within this paper and its supporting information (Figs S1–S15; Tables S1–S4).
