# Supplementary material for: GS2 cooperates with IPA1 to control panicle architecture
Source: New Phytol. 2025 Jan 31;245(6):2726–43. doi: 10.1111/nph.20412 (PMC11840411; doi:10.1111/nph.20412)
Supplement: Supplementary file 1 — Fig. S1 Different mutation forms of GS2, IPA1 and DEP1. Fig. S2 The self‐activation activity of GS2 and GS2™ proteins with 1–259 amino acids in yeast cells. Fig. S3 The effect of GS2 on phenotypes in ZH11, GS2™, GS2™‐KO and GS2‐KO plants. Fig. S4 Phenotype analysis of ZH11, GS2™, GS2™–KO, GS2‐KO and GS2‐OE plants. Fig. S5 Analysis of the cell size of ZH11, GS2™, GS2™–Ri and GS2™–OE grains. Fig. S6 The effect of the GS2 on phenotypes of ZH11, GS2™, GS2™–Ri and GS2™–OE plants. Fig. S7 Stem traits of GS2™ and GS2™‐Ri. Fig. S8 The expression of OsmiR396c. Fig. S9 The Interactions between GS2, GS2™ and IPA1™1, IPA1™2 proteins. Fig. S10 GS2 regulates the expression of DEP1. Fig. S11 GS2™ regulates the expression of DEP1. Fig. S12 GS2 regulates the expression of SRS3 and LP. Fig. S13 GS2 regulates the expression of LG1 and FZP. Fig. S14 The effect of the GS2, IPA1 and DEP1 on phenotypes of 9311 and WY plants. Fig. S15 The expression of FZP, LG1, LP and SRS3. [file NPH-245-2726-s002.pdf]

***New Phytologist* Supporting Information**

Article title: GS2 cooperates with IPA1 to control panicle architecture

Authors: Yueying Wang [1†], Yang Lv [1†], Yi Wen [1†], Junge Wang [1], Peng Hu [1], Kaixiong Wu [1], Bingze Chai [1], Shuxian Gan [2], Jialong Liu [1], Yue Wu [1], Lixin Zhu [1], Nannan Dong[1], Yiqing Tan [1], Hao Wu [1], Guangheng Zhang [1,3], Li Zhu [1], Deyong Ren [1], Qiang Zhang [1,3], Yuexing Wang [1*], Qian Qian [1,3*] and Jiang Hu [1,3,4*]

1 State Key Laboratory of Rice Biology and Breeding, China National Rice Research Institute, Hangzhou, 311401, China

2 Institute of Agricultural Sciences, Xishuangbanna Prefecture, Jinghong, Yunnan Province, 666100, China

3 Academician Workstation, National Nanfan Research Institute (Sanya), Chinese Academy of Agricultural Sciences, Sanya, 572024, China

4 Hainan Seed Industry Laboratory, Sanya 572024, China

[†] These authors contributed equally to this work.

[*] Authors for correspondence. Email: wangyuexing@caas.cn, qianiqn188@hotmail.com, hujiang588@163.com

Article acceptance date: 4 January 2025

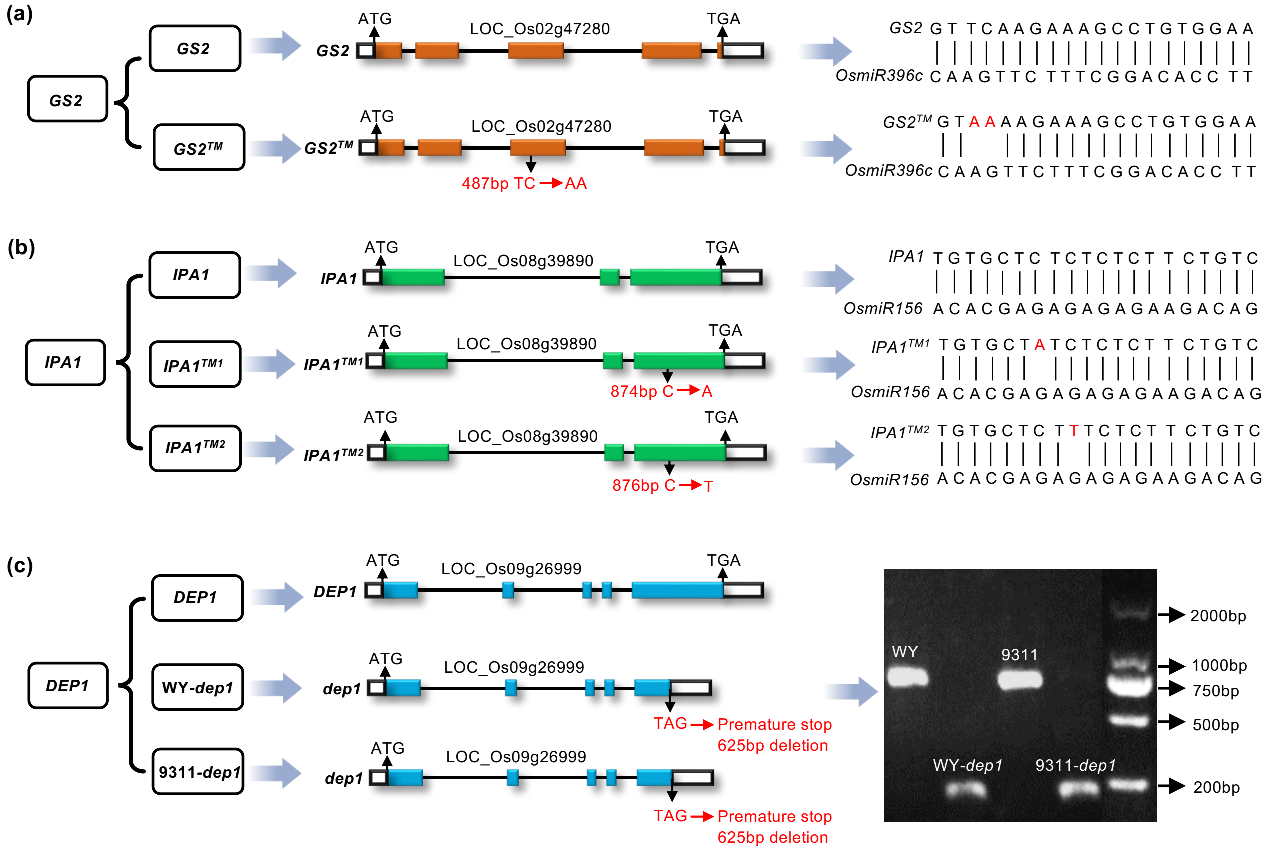

Fig. S1 Different mutation forms of *GS2*, *IPA1* and *DEP1*.

(a) Gene structure and mutation sites of *GS2* and *GS2^{TM}*, and schematic diagram of *GS2*, *GS2^{TM}* and *OsmiR396c*. (b) Gene structure and mutation sites of *IPA1*, *IPA1^{TM1}* and *IPA1^{TM2}*, and schematic diagram of *IPA1*, *IPA1^{TM1}*, *IPA1^{TM2}* and *OsmiR156*. (c) Gene structure and mutation sites of *DEP1,* WY-*dep1* and 9311-*dep1*, and the agarose gel electrophoresis results of WY, WY-*dep1*, 9311 and 9311-*dep1*. Empty boxes indicate the 5' UTR and 3' UTR, ATG and TGA indicate the start codon and stop codon, brown and green boxes indicate exons, and the line between the brown and green boxes indicate introns.

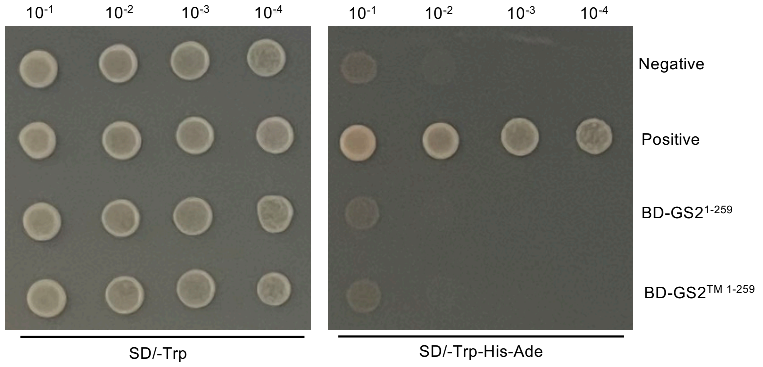

Fig. S2 The self-activation activity of GS2 and GS2^{TM} proteins with 1-259 amino acids in yeast cells.

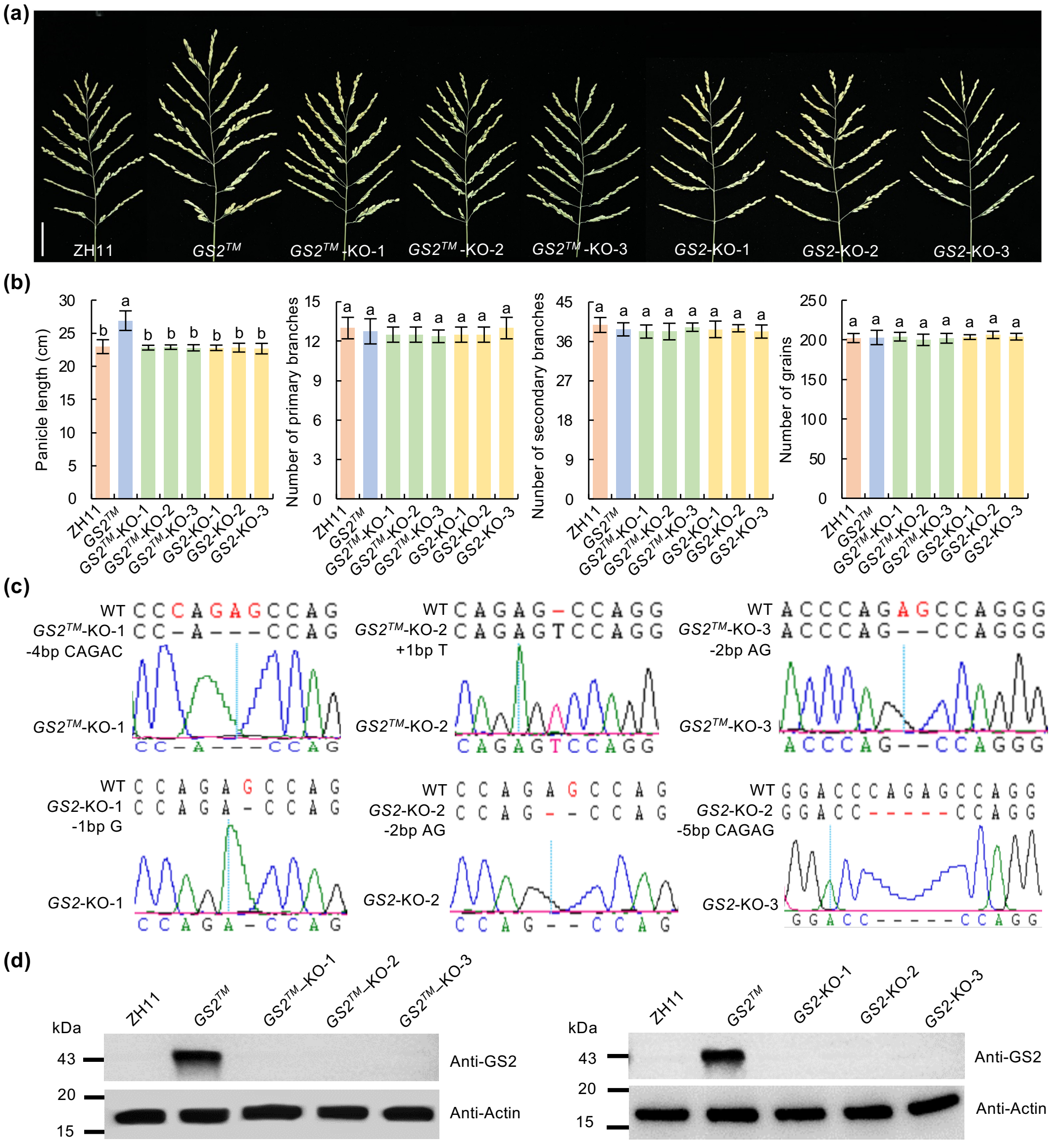

Fig. S3 The effect of *GS2* on phenotypes in ZH11, *GS2^TM^*, *GS2^TM^*-KO and *GS2*-KO plants.

(a) Panicle phenotypes of ZH11, *GS2^TM^*, *GS2^TM^*-KO and *GS2*-KO plants. Scale bar, 5 cm. (b) Panicle length, number of primary and secondary branches and number of grains in the main panicle (n = 20). (c) Mutant forms of *GS2^TM^*-KO and *GS2*-KO transgenic plants. (d) The protein expression of GS2. The protein was extracted from the 5-10 cm young panicle of ZH11, *GS2^TM^*, *GS2^TM^*-KO and *GS2*-KO plants. Data are shown as means ± standard deviation (*SD*) . One-way ANOVA, letters indicate significant differences, $P < 0.05$.

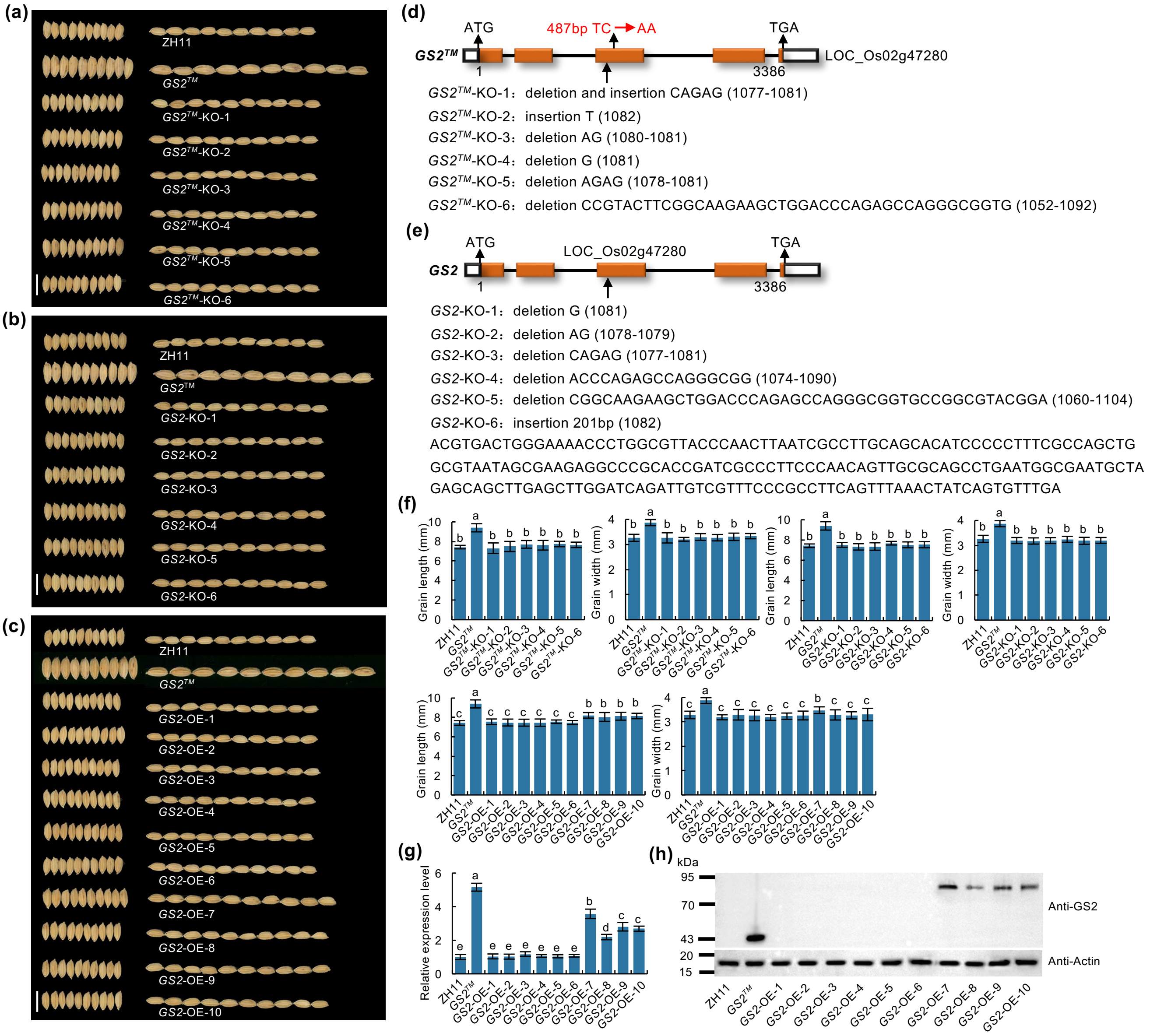

Fig. S4 Phenotype analysis of ZH11, *GS2*^{TM}, *GS2*^{TM}-KO, *GS2*-KO and *GS2*-OE plants.

(a-c) Parental grain phenotypes of ZH11, *GS2*^{TM}, *GS2*^{TM}-KO (a), *GS2*-KO (b) and *GS2*-OE (c, *proUbi-GS2-GFP-Flag*). Scale bar, 1 cm. (d, e) Mutant forms of *GS2*^{TM}-KO (d) and *GS2*-KO (e) transgenic plants. (f) Grain length and width of ZH11, *GS2*^{TM}, *GS2*^{TM}-KO, *GS2*-KO and *GS2*-OE grains (n = 20). (g) The RNA expression of *GS2*. RNA was extracted from the 5-10 cm young panicle of ZH11 and *GS2*-OE plants. (h) The protein expression levels of GS2. Data are shown as means ± standard deviation (*SD*) . One-way ANOVA, letters indicate significant differences, $P < 0.05$.

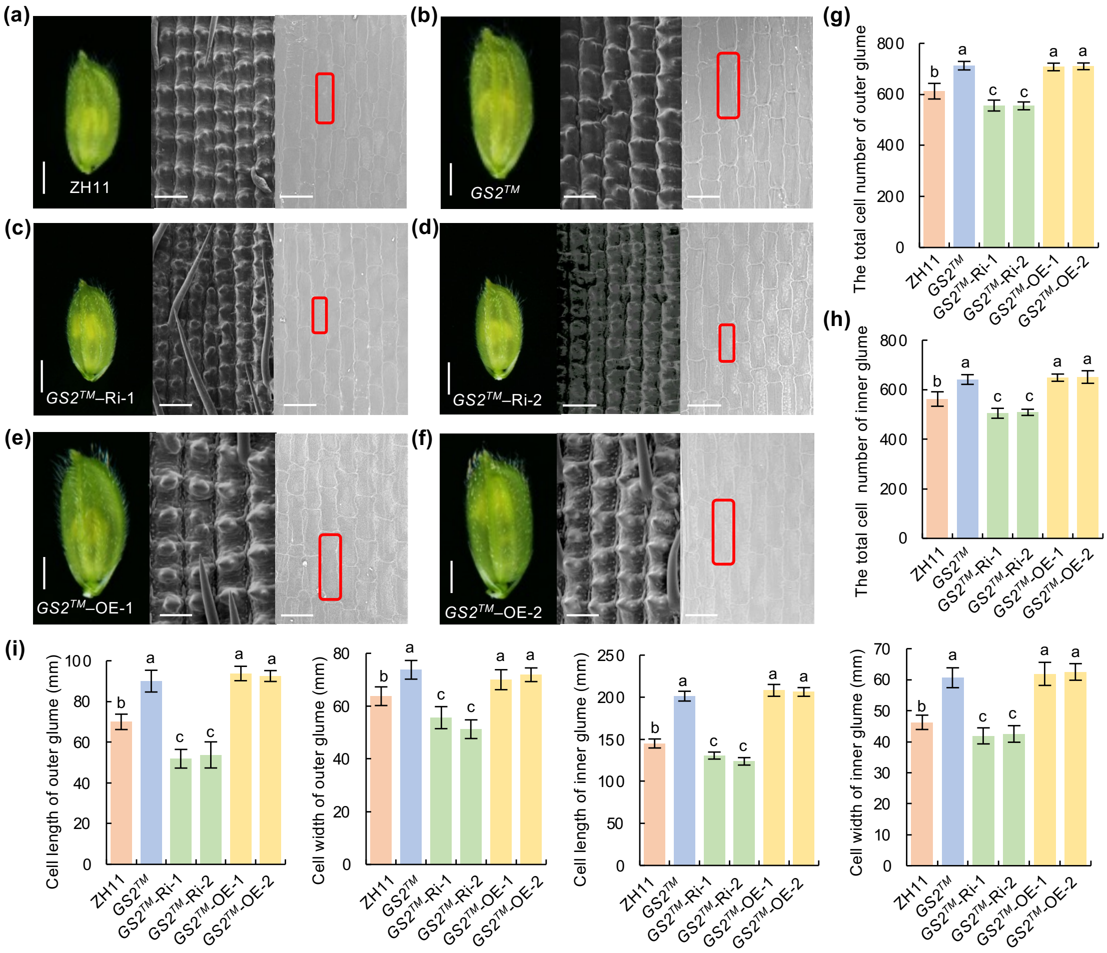

Fig. S5 Analysis of the cell size of ZH11, *GS2*^{TM}, *GS2*^{TM}-Ri and *GS2*^{TM}-OE grains.

(a-f) Cell size of spikelet hulls of ZH11, *GS2*^{TM}, *GS2*^{TM}-Ri and *GS2*^{TM}-OE. Scale bar, 2 mm in spikelet hulls. Scale bar, 100 μm for cells. (g-i) Histological analysis of spikelet hulls ZH11, *GS2*^{TM}, *GS2*^{TM}-Ri and *GS2*^{TM}-OE. Data are shown as means ± standard deviation (*SD*) . One-way ANOVA, letters indicate significant differences, $P < 0.05$.

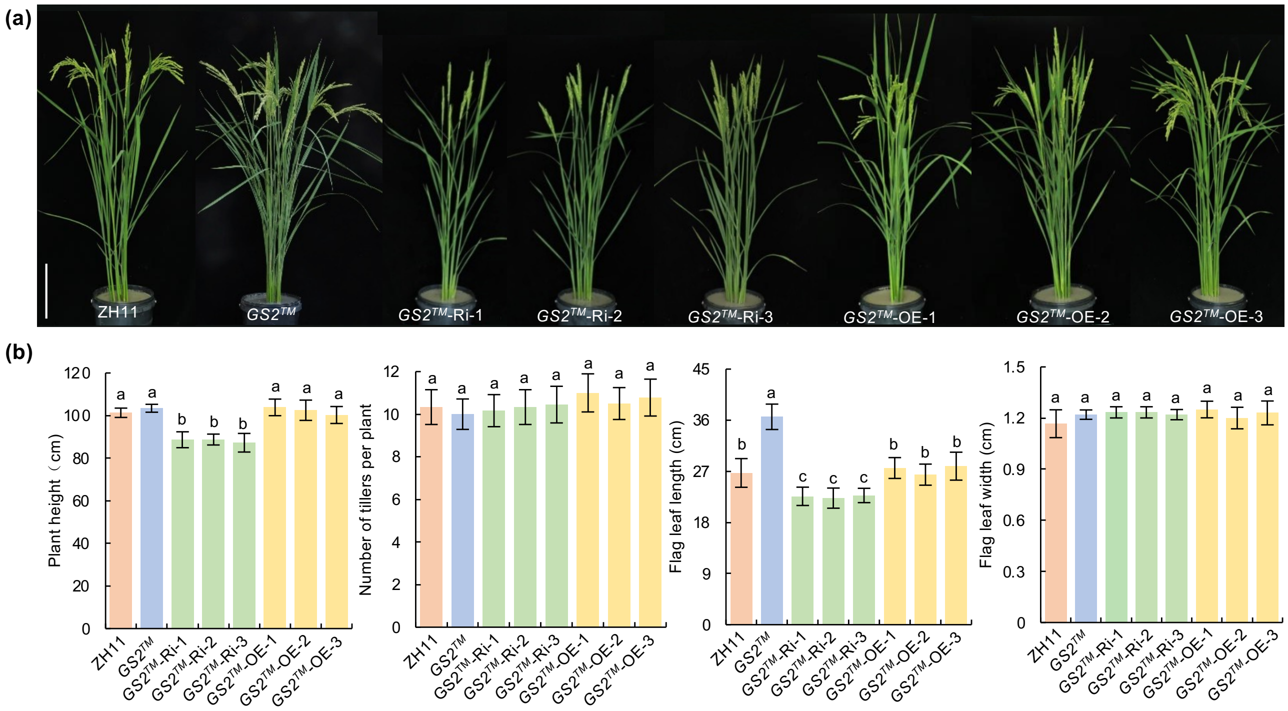

Fig. S6 The effect of the *GS2* on phenotypes of ZH11, *GS2^{TM}*, *GS2^{TM}*-Ri and *GS2^{TM}*-OE plants.

(a) Gross morphology of ZH11, *GS2^{TM}*, *GS2^{TM}*-Ri and *GS2^{TM}*-OE plants. Scale bar, 20 cm. (b) Comparison of phenotypic traits in ZH11, *GS2^{TM}*, *GS2^{TM}*-Ri and *GS2^{TM}*-OE plants (n=20). Data are shown as means ± standard deviation (*SD*) . One-way ANOVA, letters indicate significant differences, $P < 0.05$.

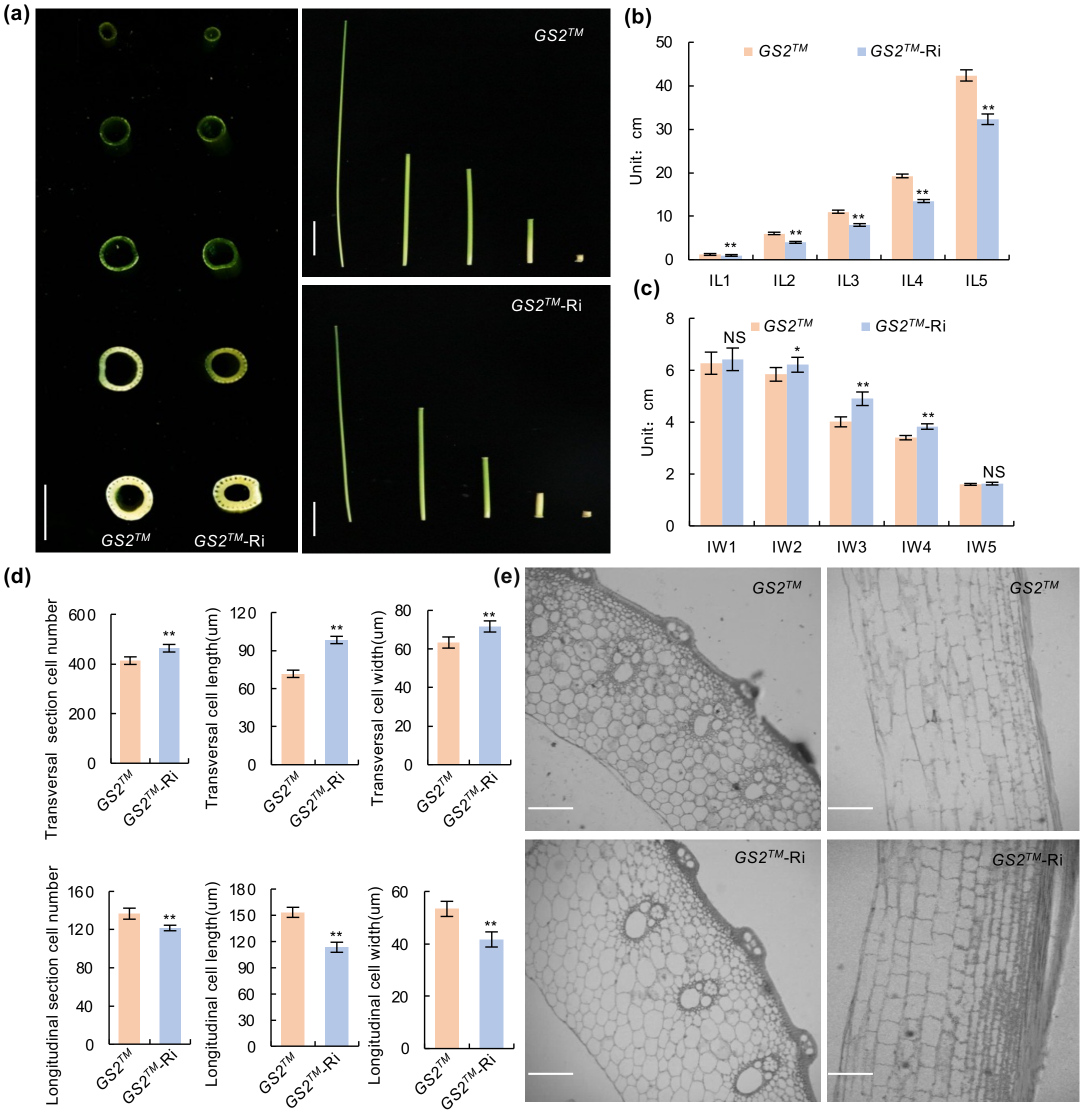

Fig. S7 Stem traits of *GS2*^{TM} and *GS2*^{TM}-Ri .

(a) Transversal and longitudinal section of stem of *GS2*^{TM} and *GS2*^{TM}-Ri plants. Scale bar, 1 cm and 2 cm in transversal and longitudinal section of stem, respectively. (b, c) Comparison of internode length and width in *GS2*^{TM} and *GS2*^{TM}-Ri plants (n = 3). (d) Comparison analysis of cell length and width in transversal and longitudinal section of stem (n = 3). (e) Paraffin transversal and longitudinal section of stem of *GS2*^{TM} and *GS2*^{TM}-Ri plants. Scale bar, 800 μm. Data are given as means ± standard deviation (*SD*) . Student's *t*-test was used to generate the *P* values; $^{**}P < 0.01$

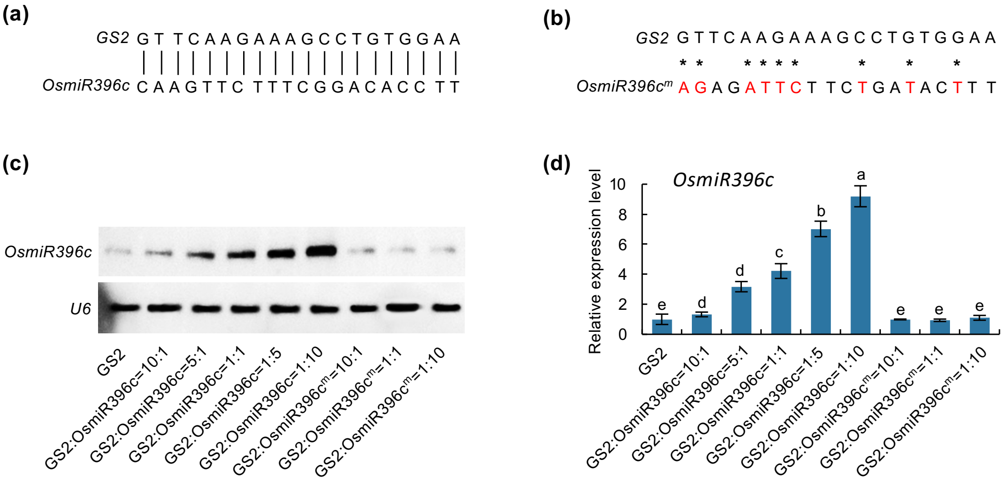

Fig. S8 The expression of *OsmiR396c*.

(a) The schematic diagram of *GS2* and *OsmiR396c*. (b) The schematic diagram of *GS2* and *OsmiR396c*^{m}. (c) Northern blotting showing the accumulation of *OsmiR396c* in rice protoplasts co-transformed with *GS2* and *OsmiR396c*, with U6 as loading control. (d) The RNA expression of *OsmiR396c*. RNA was extracted from rice protoplasts co-transformed with *GS2* and *OsmiR396c*. Data are shown as means ± standard deviation (*SD*) (n=3). One-way ANOVA, letters indicate significant differences, $P < 0.05$.

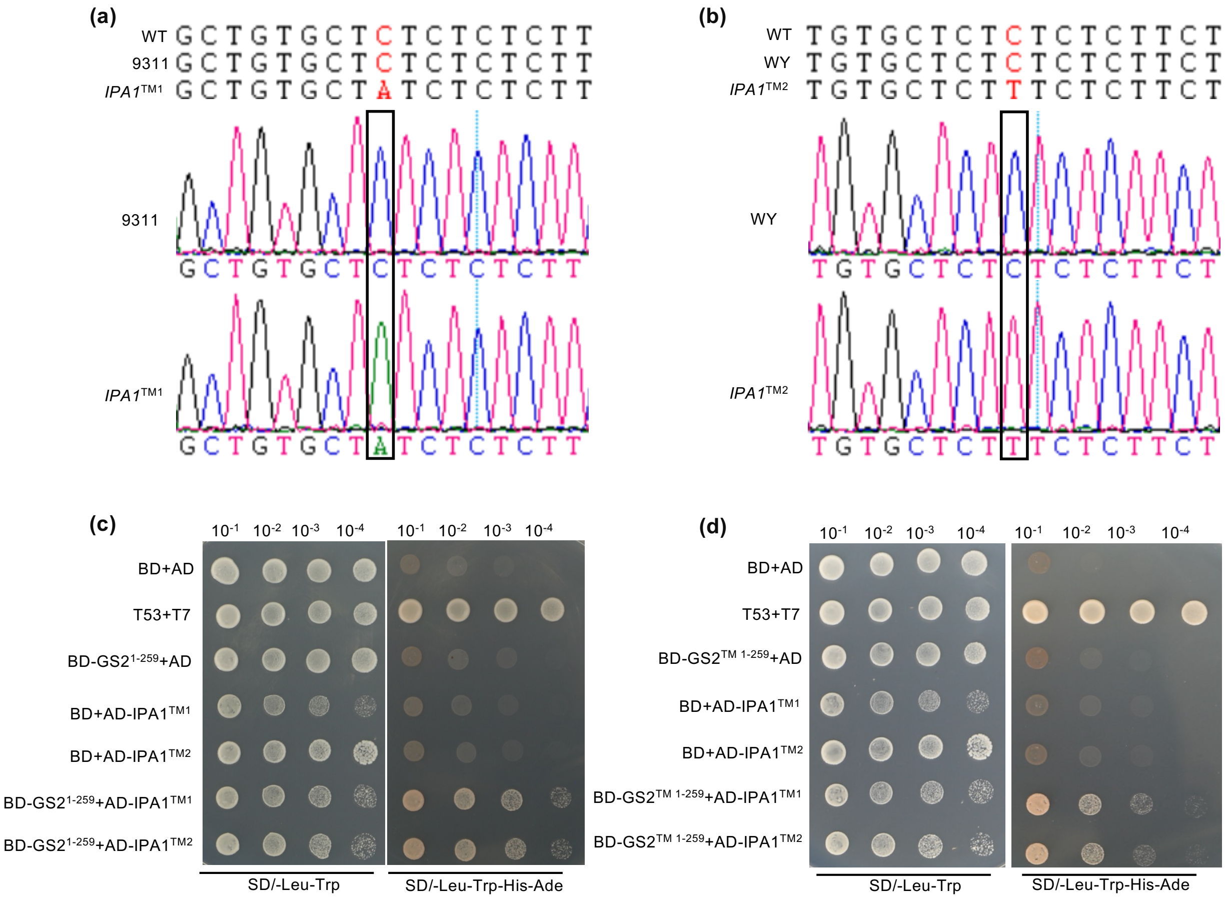

Fig. S9 The Interactions between GS2, GS2^{TM} and IPA1^{TM1}, IPA1^{TM2} proteins.

(a) Mutant forms of 9311 and *IPA1^{TM1}* plants. (b) Mutant forms of WY and *IPA1^{TM2}* plants. (c) Interaction analysis between GS2 and IPA1^{TM1}, IPA1^{TM2} in yeast two-hybrid assay. (d) Interaction between GS2^{TM} and IPA1^{TM1}, IPA1^{TM2} in yeast two-hybrid assay.

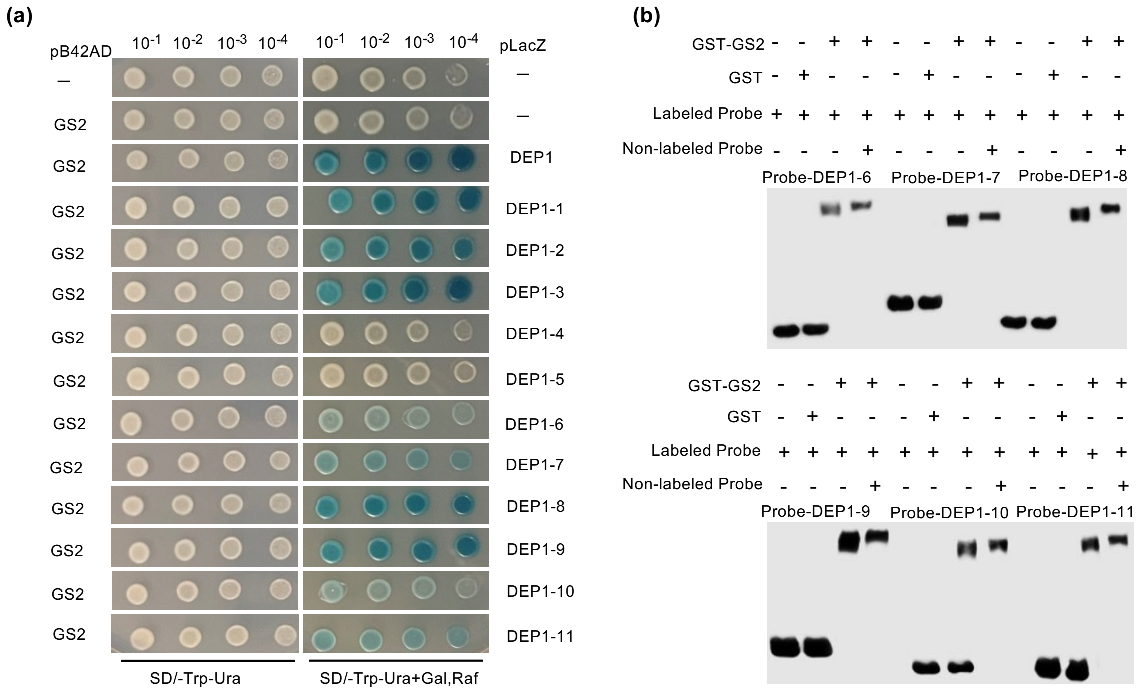

Fig. S10 GS2 regulates the expression of *DEP1*.

(a) Y1H analysis of the interaction between GS2 and *DEP1*. (b) EMSA assays. GS2 can directly bind to the DNA probes of *DEP1*.

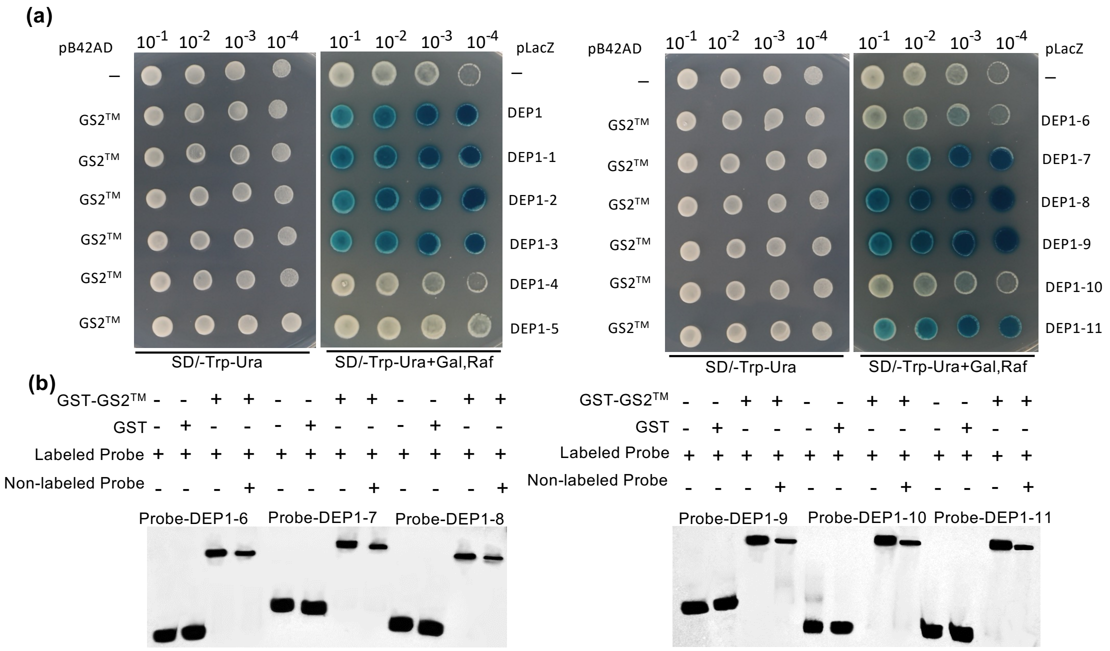

Fig. S11 GS2^{TM} regulates the expression of *DEP1*.

(a) Y1H analysis of the interaction between GS2^{TM} and *DEP1*. (b) EMSA assays. GS2^{TM} can directly bind to the DNA probes of *DEP1*.

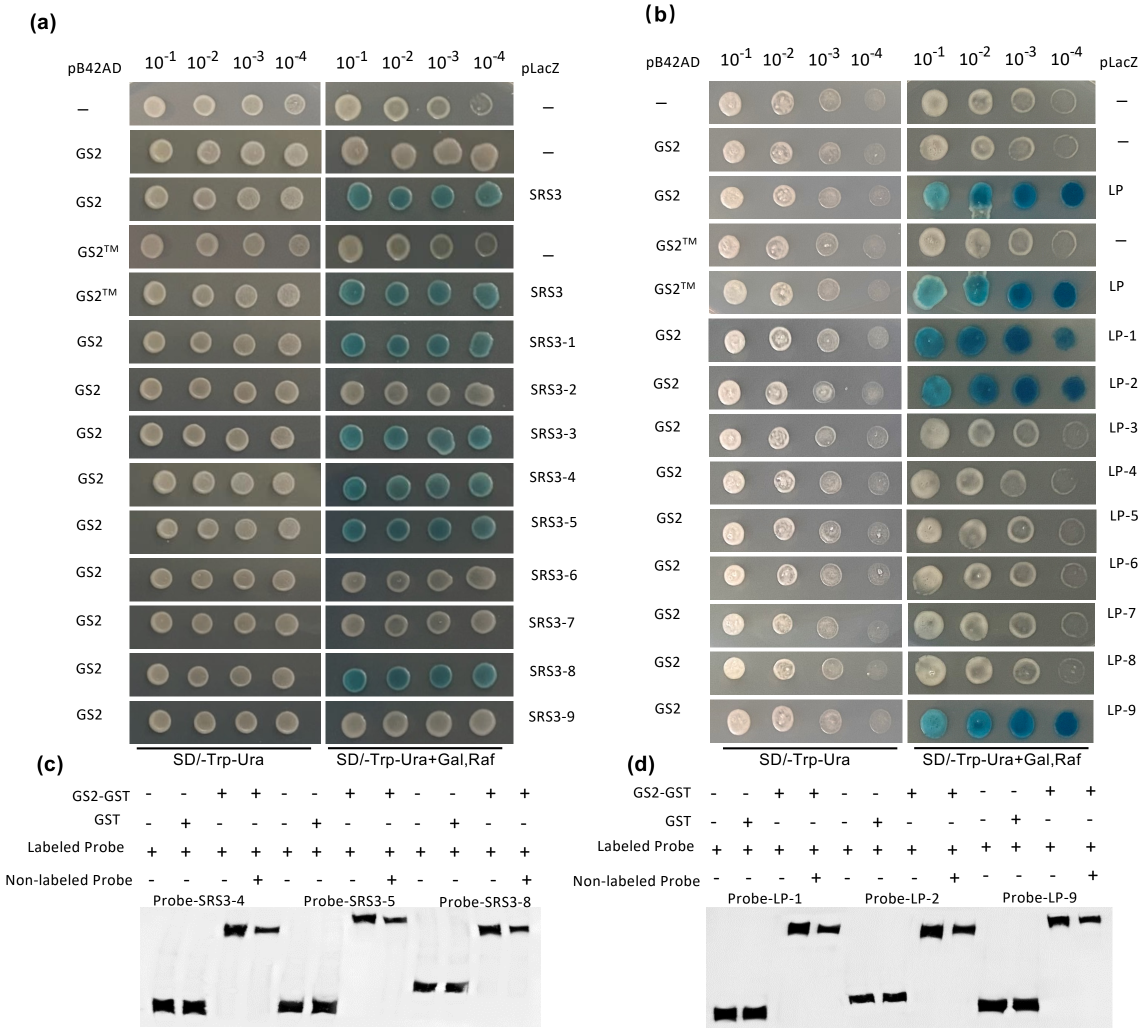

Fig. S12 *GS2* regulates the expression of *SRS3* and *LP*.

(a, b) Y1H analysis of the interaction between *GS2* and *SRS3* or *LP*. (c, d) EMSA assays. *GS2* can directly bind to the DNA probes.

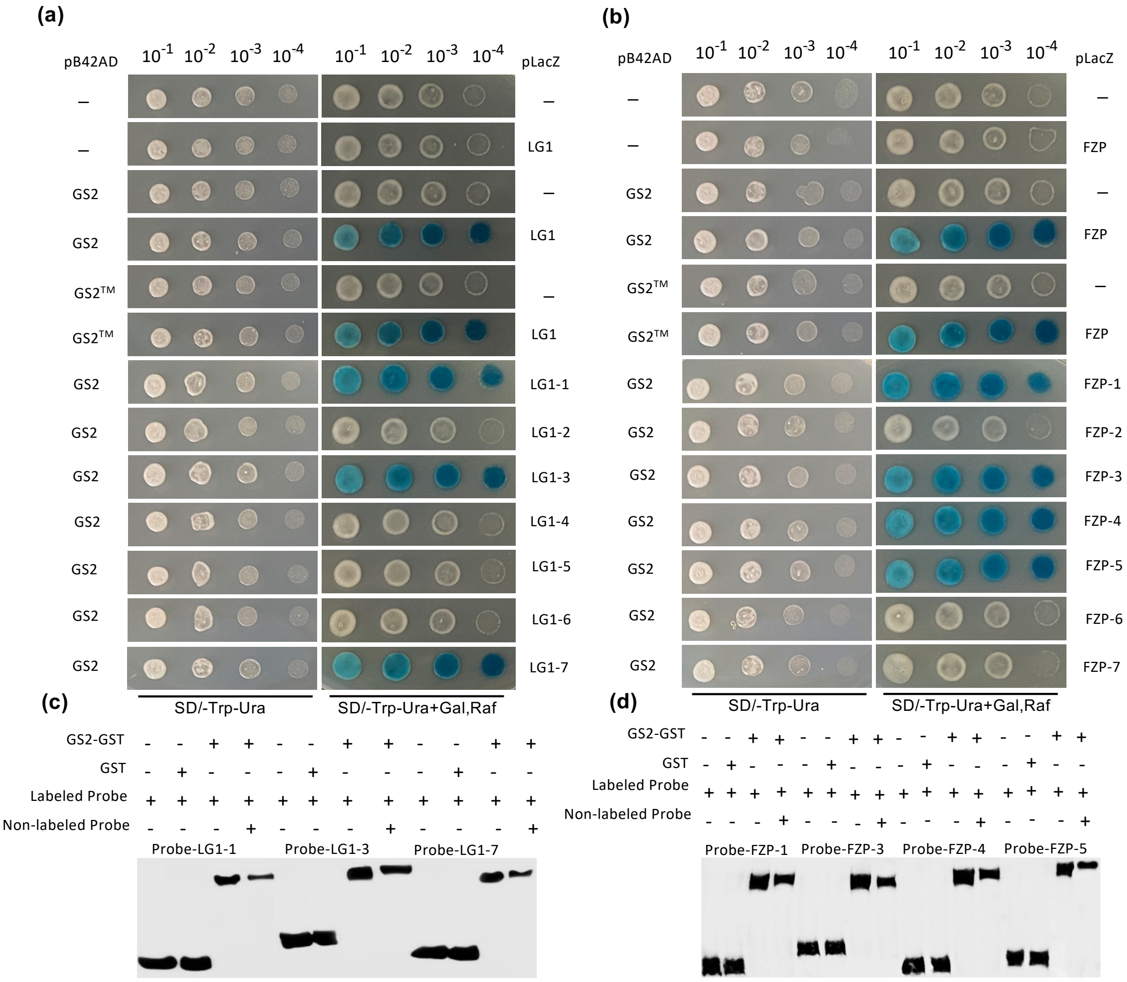

Fig. S13 *GS2* regulates the expression of *LG1* and *FZP*.

(a, b) Y1H analysis of the interaction between *GS2* and *LG1* or *FZP*. (c, d) EMSA assays. *GS2* can directly bind to the DNA probes.

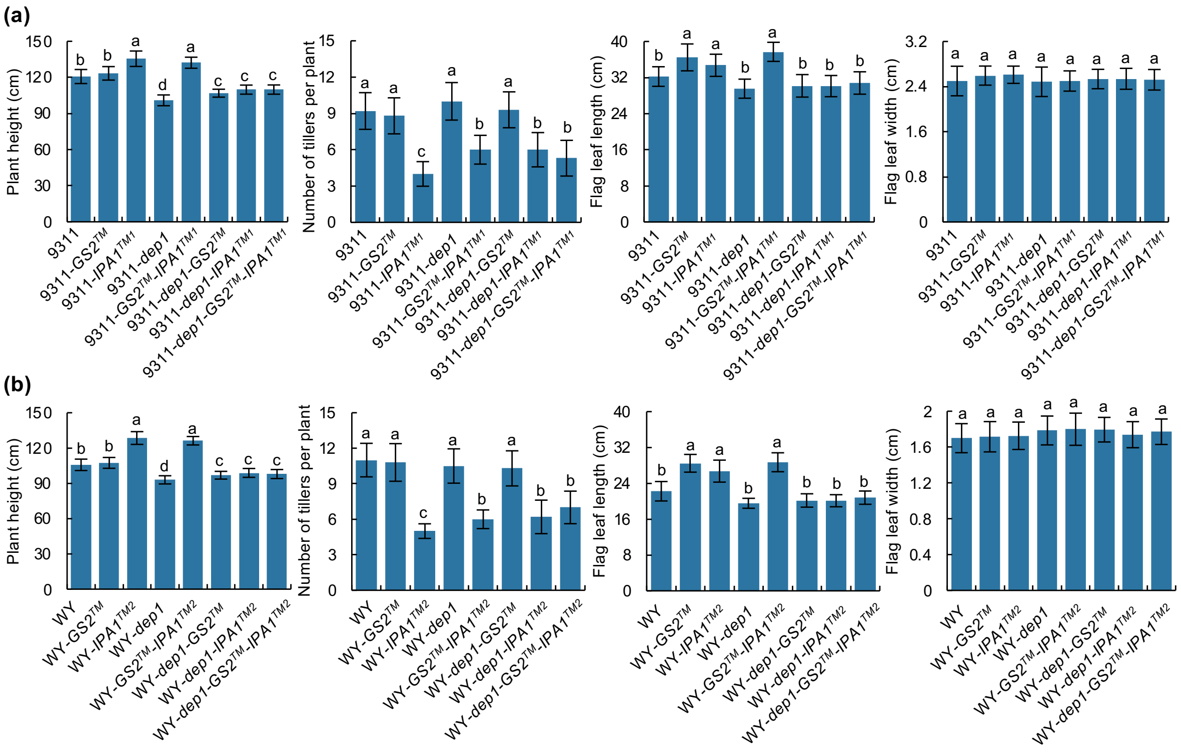

Fig. S14 The effect of the *GS2*, *IPA1* and *DEP1* on phenotypes of 9311 and WY plants.

(a, b) Plant height, number of tillers per plant, flag leaf length and flag leaf width of genetic materials of *GS2*, *IPA1* and *DEP1* in the background of 9311 (a) and WY (b) plants (n = 20). Data are shown as means ± standard deviation (*SD*) . One-way ANOVA, letters indicate significant differences, $P < 0.05$.

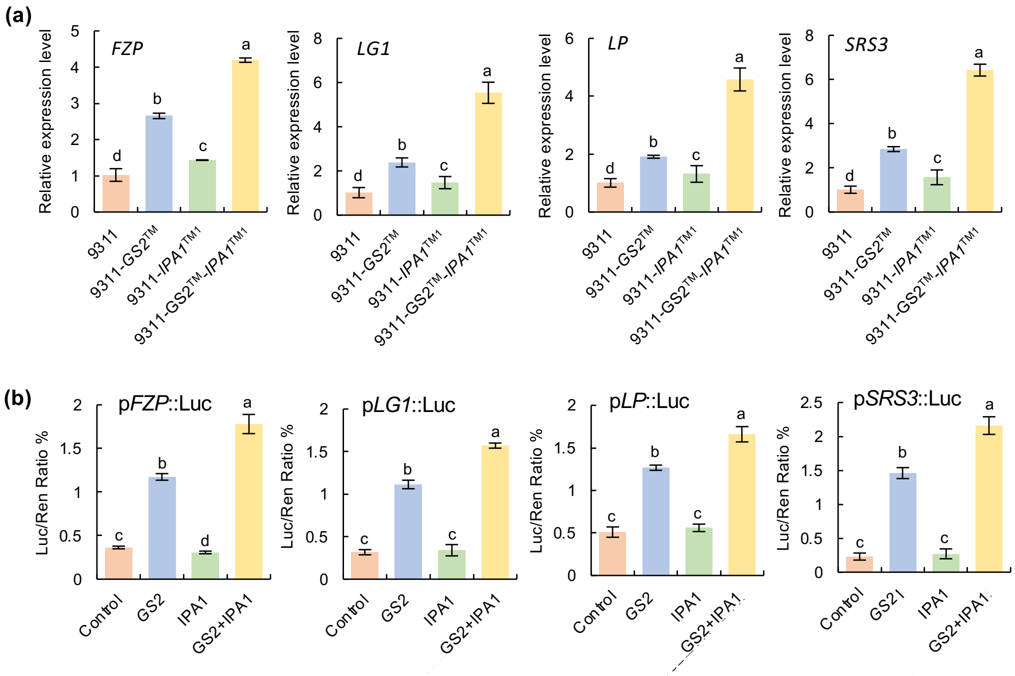

Fig.S15 The expression of *FZP*, *LG1*, *LP* and *SRS3*.

(a) The expression of *FZP*, *LG1*, *LP* and *SRS3* in 5-10 cm young panicles of 9311, 9311-*GS2^{TM}*, 9311-*IPA1^{TM1}* and 9311-*GS2^{TM}*-*IPA1^{TM1}*. (b) LUC transient transactivation assay in rice protoplasts. Data are shown as means ± standard deviation (*SD*) . One-way ANOVA, letters indicate significant differences, $P < 0.05$.
